# Saffron-Derived Antioxidant and *Passiflora incarnata* L. Extract Preserve Cerebellar Fetuin-A and Protect Purkinje Cells Against MPTP/MPP^+^-Induced Injury

**DOI:** 10.3390/antiox15101319

**Published:** 2026-10-13

**Authors:** Seohee Choi, Chae-Young Kim, Kayoung Ko, Miri Jo, Jinhye Kim, Isaac Jinwon Yi, Sang-Soep Nahm, Kiyoung Kim, Sun-Shin Yi

**Affiliations:** 1 BK21 Four Program, Department of Medical Sciences, Soonchunhyang University, Asan 31538, Republic of Korea; suhhee1021@naver.com (S.C.); gurqls0404@naver.com (K.K.); jml2213@naver.com (M.J.); kiyoung2@sch.ac.kr (K.K.); 2 Department of Anatomy, College of Veterinary Medicine, Konkuk University, Seoul 05029, Republic of Korea; kcybada@gmail.com (C.-Y.K.); snahm@konkuk.ac.kr (S.-S.N.); 3 Central Lab, iCONNECTOME Co., Ltd., Suwon 16229, Republic of Korea; adjh08@iconnectome.com; 4 Neuroscience and Behavioral Biology Program (NBB), College of Arts and Sciences, Emory University, Atlanta, GA 30322, USA; jinwon.yi@emory.edu

**Keywords:** Parkinson’s disease, oxidative stress, reactive oxygen species, Fetuin-A, Purkinje cell, cerebellum, saffron, *Passiflora incarnata*, MPTP, neuroprotection

## Abstract

Oxidative stress contributes to neuronal injury in Parkinson’s disease (PD), and the cerebellum is increasingly recognized as an affected region. We previously reported that Fetuin-A (alpha-2-HS-glycoprotein) is reduced in cerebellar Purkinje cells in human PD and in 1-methyl-4-phenyl-1,2,3,6-tetrahydropyridine (MPTP)-treated mice and that silencing it reduces Purkinje cell survival without apoptosis. We asked whether this reduction is pharmacologically modifiable by saffron-derived antioxidant (SDA) and *Passiflora incarnata* L. extract (PIE). SDA and PIE were administered orally at 50 mg/kg/day for 5 weeks to MPTP-treated C57BL/6J mice, with resveratrol (RES) as a comparator. In 1-methyl-4-phenylpyridinium (MPP^+^)-challenged primary cerebellar neurons, SDA and PIE were tested at 25 µg/mL and RES at 200 µg/mL. MPTP lowered nigral tyrosine hydroxylase immunoreactivity, grip strength, rotarod performance, Purkinje cell density and cerebellar Fetuin-A and raised cleaved caspase-3, terminal deoxynucleotidyl transferase dUTP nick-end labeling (TUNEL) and Fluoro-Jade C signals. SDA and PIE attenuated all these changes. In vitro, MPP^+^ raised 2′,7′-dichlorofluorescin diacetate (DCFDA) fluorescence and lowered Fetuin-A, and all three treatments reduced DCFDA fluorescence and restored Fetuin-A. Annexin V/propidium iodide (PI) profiling showed a substantial Annexin V^−^/PI^+^ necrotic component at 24 h, consistent with our earlier knockdown data. Cerebellar Fetuin-A emerges as a treatment-responsive correlate of Purkinje cell integrity.

## 1. Introduction

Parkinson’s disease (PD) is a progressive neurodegenerative disorder defined clinically by motor and non-motor manifestations and pathologically by degeneration of dopaminergic neurons in the substantia nigra pars compacta (SNc) [1,2]. Although the nigrostriatal system remains central to PD, oxidative stress, mitochondrial dysfunction and neuroinflammation form an interconnected network that determines which neuronal populations survive [3,4]. Redox imbalance is not merely a downstream consequence of dopaminergic degeneration. Neurons with high tonic firing rates and heavy calcium-handling loads operate with a narrow antioxidant reserve, and interventions that either scavenge reactive oxygen species (ROS) or reinforce endogenous antioxidant programs remain among the most actively pursued neuroprotective strategies in experimental PD [3,4,5].

1-Methyl-4-phenyl-1,2,3,6-tetrahydropyridine (MPTP) is converted in the brain to 1-methyl-4-phenylpyridinium (MPP^+^), which is concentrated by the dopamine transporter and inhibits mitochondrial complex I, generating superoxide and depleting ATP [1]. Because complex I inhibition is fundamentally a redox lesion and because repeated MPTP exposure sustains neuroinflammation over weeks [6], the chronic MPTP/MPP^+^ paradigm provides a mechanistically appropriate platform for testing antioxidant interventions.

The cerebellum is increasingly recognized as part of the network affected in PD. Imaging, neurophysiological and pathological evidence indicates that cerebellar alterations reflect both compensatory responses and disease-associated dysfunction [7,8,9], and MPTP has been shown to alter calcium-binding protein expression and the firing behavior of Purkinje cells in primates [10,11]. Purkinje cells are the sole output neurons of the cerebellar cortex. Their extensive dendritic arbors, tonic high-frequency firing and calcium-dependent signaling impose an exceptional metabolic and redox burden that plausibly underlies their vulnerability [12].

In our previous study, we demonstrated, in cerebellar tissue from patients with PD and in MPTP-treated mice, that Fetuin-A (alpha-2-HS-glycoprotein, *AHSG*) was markedly reduced in Purkinje cells and that small interfering RNA-mediated silencing of Fetuin-A in embryonic day 18 (E18) cerebellar neurons reduced the number of Calbindin-positive cells [13]. Notably, that reduction in cell number occurred without a corresponding increase in TUNEL positivity, indicating that Fetuin-A loss compromises Purkinje cell survival through a mechanism that is not classical apoptosis [13]. Fetuin-A is a multifunctional glycoprotein with systemic metabolic, mineral-binding and immunomodulatory functions [14,15,16]. Experimental evidence from brain-injury models supports a context-dependent cytoprotective role. Peripheral Fetuin-A administration attenuated early cerebral ischemic injury [17], Fetuin-A is redistributed in inflamed and ischemic brain tissue [18], and Fetuin-A suppressed microglial necroptosis through nuclear factor erythroid 2-related factor 2 (NRF2)/heme oxygenase-1 (HO-1) signaling after traumatic brain injury [19]. These observations link Fetuin-A to the NRF2 axis in other injury models.

In parallel work on the same experimental model, we identified an astrocytic NADPH oxidase 4 (NOX4)–myeloperoxidase (MPO)–osteopontin (OPN) axis as a driver of redox-inflammatory injury in the hippocampus, in which NOX4-derived lipid peroxidation promotes a non-apoptotic, ferroptosis-like form of cell death [20,21,22]. Both downstream mediators of this axis have independently been linked to neurodegeneration. MPO-immunoreactive cells are increased in affected brain regions in PD and Alzheimer’s disease [23], and OPN has been repeatedly implicated in neurodegenerative pathology [24]. In a separate study conducted in parallel with the present work, using the same MPTP paradigm and the same oral dosing regimen in an independent group of mice, saffron-derived antioxidant (SDA) and *Passiflora incarnata* L. extract (PIE) suppressed hippocampal NOX4, MPO and OPN, lowered the lipid-peroxidation adduct 4-hydroxynonenal (4-HNE), and restored mitochondrial import and oxidative phosphorylation proteins [25]. Those findings indicate that the two extracts influence redox-related processes in vivo in this model, and they motivated the present question of whether the same interventions protect the cerebellar Purkinje cell population and its Fetuin-A pool.

Saffron (*Crocus sativus* L.) and its apocarotenoid constituents—crocin, crocetin, picrocrocin and safranal—display direct radical-scavenging capacity and neuroprotective activity across diverse experimental settings [26,27,28,29,30,31,32,33], and a standardized saffron extract from the same supplier used here has been characterized by our group in a chronic mild stress model [34] and in the clinical literature [35,36,37]. *P. incarnata* L. contains flavone C-glycosides. The extract lineage used in the present study was standardized to vitexin and characterized by HPLC-UV and LC-MS/MS, which identified isoorientin, orientin, vitexin and isovitexin as its principal flavonoids [38,39]. The same standardized preparation conferred resistance to hydrogen-peroxide-induced oxidative stress, extended its lifespan and induced the stress-responsive *hsp-16.2* reporter in *Caenorhabditis elegans* [38], and vitexin itself protects dopaminergic neurons in MPTP-treated mice [40] and augments NRF2/HO-1-dependent antioxidant signaling [41]. Whether these preparations reach the brain is relevant to their interpretation. Crocin is poorly absorbed in its glycosylated form and is hydrolyzed in the intestine to *trans*-crocetin, which permeates intestinal and blood–brain barrier models in vitro [42]. Brain exposure to PIE and to vitexin was not measured in the present study, although the neuroprotective effect of systemically administered vitexin noted above is consistent with central exposure. Resveratrol (RES), whose neuroprotective and antioxidant activities in MPTP models are well documented [43,44,45,46,47], was included as a reference antioxidant comparator.

We, therefore, asked whether chronic MPTP/MPP^+^ exposure lowers cerebellar Fetuin-A in parallel with Purkinje cell injury, whether treatment with SDA or PIE limits both, and whether Fetuin-A preservation is accompanied by reduced intracellular 2′,7′-dichlorofluorescin diacetate (DCFDA) fluorescence in MPP^+^-challenged cerebellar neurons.

## 2. Materials and Methods

### 2.1. Test Materials and Their Prior Standardization

Resveratrol (RES; Activ’Inside, Beychac-et-Caillau, France) was included as a reference antioxidant comparator because of its established neuroprotective and antioxidant activity in MPTP-induced PD models [43,44,45,46,47]. The saffron-derived antioxidant (SDA; affron^®^; Pharmactive Biotech Products S.L., Madrid, Spain) and the *Passiflora incarnata* L. extract (PIE) were the principal test interventions. Throughout this paper, PIE denotes the *P. incarnata* L. extract, whereas PI denotes propidium iodide in the Annexin V/PI flow-cytometric analyses and the corresponding figure axis labels.

**SDA.** SDA was the standardized saffron extract affron^®^ (Pharmactive Biotech Products S.L., Madrid, Spain; batch SAF-30415-PH, product code SAF0203107). According to the supplier’s certificate of analysis, the material is prepared from *Crocus sativus* L. stigmas of Spanish origin by aqueous extraction and is supplied as a dark orange powder consisting of native extract together with dextrin as a carrier. The preparation is standardized to a proprietary marker fraction, Lepticrosalides^®^, quantified by HPLC-DAD (specification > 3.5%, with the measured value for the batch used here being 4.06%), and the batch conformed to the supplier’s specifications for identity, affron^®^ profile, bulk density (0.51 g/mL), ash (1.15%), loss on drying (2.14%) and particle size, as well as to the applicable microbiological, heavy-metal, mycotoxin and pesticide-residue limits. Because the marketed powder contains dextrin, the administered dose of 50 mg/kg/day refers to the formulated extract as supplied and not to the native extract alone. The same standardized preparation from this supplier was used by our group at 100 and 200 mg/kg/day in a chronic mild-stress rat model [34]. The pharmacology of saffron and of its apocarotenoid constituents crocin, crocetin, picrocrocin and safranal has been reviewed in detail elsewhere [26,27,37]. Individual apocarotenoids were not separately quantified in the present batch.

**PIE.** PIE was prepared from *P. incarnata* leaves and fruits (8:2, *w*/*w*), extracted with 60% aqueous ethanol, concentrated and dried, and standardized against vitexin as the reference marker, exactly as described and analytically validated in our previous work [38,39]. The same standardized extract used in the present study was supplied by Natural F&P Co., Ltd., Yongin, Republic of Korea. In our previous phytochemical characterization [38], vitexin was quantified by HPLC-UV using an Agilent HPLC system equipped with an Agilent 1260 binary pump, Agilent 1100 autosampler, and Agilent 1200 diode-array detector (Agilent Technologies, Palo Alto, CA, USA), with a Shim-pack VP-ODS C18 column (250 × 4.6 mm, 5 µm) and an isocratic mobile phase consisting of tetrahydrofuran/acetonitrile/0.05% phosphoric acid solution (20:3:77, *v*/*v*/*v*) at a flow rate of 1.0 mL/min and detection at 360 nm. Previous phytochemical characterization of this extract lineage identified isoorientin, orientin, vitexin and isovitexin, with vitexin as the principal marker compound, and established the calibration curve used for standardization [38].

**Functional antioxidant evidence for the preparations used.** Cell-free radical-scavenging assays were not repeated on the present batches. Instead, we relied on previously published functional antioxidant characterization of the same standardized preparations and of their marker constituents. For PIE, the identical standardized extract significantly increased survival of *C. elegans* under 1 mM hydrogen peroxide challenge, extended mean and maximum lifespans, and induced the oxidative stress-responsive *hsp-16.2*::GFP reporter [38]. Its principal marker vitexin protects dopaminergic neurons in MPTP-treated mice through PI3K/Akt signaling [40] and augments NRF2/HO-1-dependent antioxidant defense [41]. For SDA, the antioxidant and radical-scavenging activity of standardized saffron extracts and of crocin and crocetin has been repeatedly demonstrated, including in MPTP and lead-induced dopaminergic injury models [30,31]. Most directly, in an independent group of mice treated with the identical regimen in our parallel study, both extracts lowered the lipid-peroxidation adduct 4-HNE and suppressed the NOX4–MPO–OPN redox-inflammatory axis in brain tissue [25]. This limitation is stated explicitly in Section 4.7.

**Dosing.** RES, SDA and PIE were freshly suspended in distilled water (vehicle) and vortexed immediately before administration. Each preparation was administered once daily by oral gavage at 50 mg/kg/day using a stainless-steel gavage needle (Φ 0.9 mm in diameter × 50 mm in length) between 15:00 and 18:00, with the dosing volume adjusted to the most recent body weight. The unified dose of 50 mg/kg/day was based on prior MPTP studies in which 50 mg/kg/day RES preserved striatal dopaminergic markers [43,44,45], on in vivo work using comparable oral doses of saffron-derived compounds [31], and on our own mouse studies showing efficacy of PIE at 10 and 50 mg/kg [38]. It is identical to the regimen used in our parallel study [25].

### 2.2. Animals, Experimental Design

Forty-five male C57BL/6J mice (Jackson Laboratory, Bar Harbor, ME, USA) were purchased at 7 weeks of age, acclimatized for 1 week and were 8 weeks old and 22 ± 2 g at the start of treatment. They were housed under controlled conditions (22 ± 2 °C, 60% relative humidity, 12 h light/dark cycle) with ad libitum access to chow (2018S; Harlan, Indianapolis, IN, USA) and distilled water. After acclimatization, mice were randomly assigned to five groups (*n* = 9/group), namely, Sham (0.9% saline + distilled water), MPTP (MPTP + distilled water), MPTP+RES, MPTP+SDA and MPTP+PIE. The individual mouse was the experimental unit. Group sizes were based on our previous studies using the same model and endpoints [13,25] rather than on a formal a priori power calculation.

Vehicle or extracts were administered orally once daily for 5 weeks. Beginning 1 week after the start of oral treatment, MPTP hydrochloride (S4732; Selleck Chemicals, Houston, TX, USA) was administered intraperitoneally at 30 mg/kg/day for 4 weeks, and Sham mice received matched saline injections (Supplementary Figure S1). This repeated 4-week MPTP regimen was established in our previous study [13], in which C57BL/6J mice received 30 mg/kg/day MPTP intraperitoneally for 28 consecutive days and developed reproducible nigral dopaminergic loss, motor impairment and cerebellar Purkinje cell abnormalities. The same regimen was applied in our parallel study [25]. Prolonged rather than acute MPTP exposure was chosen deliberately, because repeated administration sustains the neuroinflammatory and redox response that the present intervention was designed to test [6]. Humane endpoints were defined a priori in the approved protocol as a loss of more than 20% of body weight relative to the pretreatment value, inappetence persisting for more than two days, or persistent signs of distress such as unresponsiveness to stimuli. Animals were observed daily, and body weight, food intake and water intake were recorded throughout the study (Supplementary Figure S2). No animal reached these endpoints, no animal died or was removed before the end of the study, and no data were excluded. Animal allocation across behavioral, histological/immunostaining, and Western blot analyses is summarized in Supplementary Table S1.

Randomization was performed by body-weight-stratified allocation using computer-generated random numbers. Investigators performing image-based quantification and densitometry were blinded to group allocation. All procedures were approved by the Institutional Animal Care and Use Committee of Soonchunhyang University (approval no. SCH24-0015; approved on 2 February 2024) and are reported in accordance with the ARRIVE 2.0 guidelines [48]. MPTP was handled and disposed of under institutional hazardous-agent procedures.

**Relationship to our parallel study.** The animals analyzed in the present study are entirely separate from those analyzed in our parallel report on the hippocampal astrocytic NOX4–MPO–OPN axis [25]. No animal, tissue sample, image or data set is shared between the two studies. The two investigations were conducted in the same laboratory under the same institutional approval and used the same mouse strain and supplier, the same five-group design, repeated MPTP dosing regimen and the same oral dosing schedule for RES, SDA and PIE, but they were performed on independent groups of mice and examined different brain regions, the hippocampus in [25] and the cerebellum here. Every value, micrograph and immunoblot presented in this paper, including the nigral tyrosine hydroxylase images, the behavioral measurements and the body-weight, food-intake and water-intake records (Supplementary Figure S2), was obtained from the animals in the present study. The substantia nigra and the behavioral battery appear in both reports because each study assessed them independently, in each case solely to verify that the dopaminergic lesion and the associated motor deficit had been established before the region-specific analyses were undertaken. No cerebellar, Fetuin-A or primary cerebellar culture data have been previously published.

### 2.3. Behavioral Assessment

Forelimb strength was measured with a grip strength meter (Jeung Do Bio & Plant Co., Ltd., Seoul, Republic of Korea). Each mouse grasped a horizontal bar attached to a force gauge, and the peak force during gentle tail traction was recorded. Motor coordination was assessed on a rotarod (CASSCALE, Seoul, Republic of Korea) at a constant 20 rpm, with latency to fall recorded up to 300 s. Neuromuscular endurance was evaluated using an inverted wire cage lid suspended 50 cm above a cushioned surface, with latency to fall recorded up to 500 s. Before formal testing, each mouse underwent one training session per assay, consisting of 300 s on the rotarod at 4 rpm, three consecutive grip strength measurements, and one wire hang trial of up to 500 s. Each test was then performed in three trials per mouse, separated by 5 min for grip strength and rotarod and by 10 min for the wire hang, and the mean of the three trials was used for analysis. All behavioral assessments were performed after completion of treatment and before sacrifice.

### 2.4. Tissue Preparation

Mice were deeply anesthetized with urethane (20%, 10 mL/kg, i.p.; Daejung Chemicals & Metals, Siheung, Republic of Korea). Animals assigned to histology and immunostaining (*n* = 4/group) were transcardially perfused with 0.9% saline followed by 4% paraformaldehyde (P6148; Sigma-Aldrich, St. Louis, MO, USA) in phosphate-buffered saline (pH 7.4). Brains were post-fixed overnight at 4 °C, dehydrated, cleared and paraffin-embedded. Serial coronal sections (4 µm) were cut on a rotary microtome (Epredia, Kalamazoo, MI, USA). Sections corresponding to the Purkinje cell layer of the cerebellum (bregma −5.80 to −6.24 mm) and substantia nigra pars compacta (SNc) (bregma −2.70 to −2.92 mm) were selected according to the mouse brain atlas [49], as in our previous cerebellar study [13]. For Western blotting (*n* = 5/group), mice were perfused with saline only. The cerebellum was rapidly dissected, snap-frozen in liquid nitrogen and stored at −80 °C.

### 2.5. Histological and Immunostaining Analyses

**Sampling and quantification.** For each animal, three 4 µm thick coronal sections were analyzed. Cerebellar analyses were performed in the Purkinje cell layer, whereas substantia nigra analyses were restricted to the substantia nigra pars compacta (SNc), within the bregma ranges specified in Section 2.4.

**Nissl staining.** Paraffin sections of cerebellum and substantia nigra were deparaffinized, rehydrated, stained with 0.1% cresyl violet (C5042; Sigma-Aldrich, St. Louis, MO, USA), differentiated in ethanol, dehydrated, cleared and mounted with Eukitt^®^ Quick-hardening medium (03989; Sigma-Aldrich, St. Louis, MO, USA). Brightfield images were acquired with a Motic EasyScan One slide scanner (Motic, Xiamen, China). Substantia nigra images are presented at 4× scanner magnification, whereas cerebellar Purkinje-cell images are presented at 100× for the overview images and 400× for the higher-magnification images. Nissl-positive neurons in the substantia nigra were counted per unit area (cells/mm^2^) and Purkinje cells per unit length of the Purkinje cell layer (cells/mm).

**Immunohistochemistry.** Antibodies against tyrosine hydroxylase (TH; ab6621; rabbit polyclonal; 1:500; Abcam, Cambridge, UK) and cleaved caspase-3 (CC3; #9661; rabbit monoclonal; 1:200; Cell Signaling Technology, Danvers, MA, USA) were used in the substantia nigra and cerebellum, respectively. Sections were incubated with biotinylated goat anti-rabbit IgG (H+L) (BA-1000; Vector Laboratories, Burlingame, CA, USA), amplified with a VECTASTAIN® ABC-HRP kit (PK-6100; Vector Laboratories, Burlingame, CA, USA) and visualized with 3,3′-diaminobenzidine (D5637; Sigma-Aldrich, St. Louis, MO, USA). Sections processed in parallel with omission of the primary antibody served as negative controls. Brightfield images were acquired with the Motic EasyScan One slide scanner (Motic, Xiamen, China). TH images are presented at 6× scanner magnification, whereas cleaved caspase-3 images are presented at 100× and 250× scanner magnifications for the overview and higher-magnification images, respectively. TH-positive cells were quantified per unit area and CC3-positive cells per unit length of the Purkinje cell layer.

**Immunofluorescence.** Cerebellar sections were incubated with anti-Fetuin-A (SC-133146; mouse monoclonal; 1:50; Santa Cruz Biotechnology, Dallas, TX, USA) and anti-Calbindin D-28k (CB-38a; rabbit polyclonal; 1:500; Swant, Burgdorf, Switzerland). To minimize background associated with the use of a mouse primary antibody on mouse tissue, sections were processed using the M.O.M. (Mouse on Mouse) Immunodetection Kit, Basic (BMK-2202; Vector Laboratories, Burlingame, CA, USA), according to the manufacturer’s instructions. Sections were then incubated with donkey anti-mouse IgG (H+L) Alexa Fluor™ 488 (A21202; Invitrogen, Carlsbad, CA, USA) and donkey anti-rabbit IgG (H+L) Alexa Fluor™ 594 (A21207; Invitrogen, Carlsbad, CA, USA), each matched to the host species of the corresponding primary antibody. This antibody combination is the one validated for cerebellar Fetuin-A/Calbindin double labeling in our previous study [13]. Nuclei were counterstained with VECTASHIELD Antifade Mounting Medium with DAPI (H-1200-10; Vector Laboratories, Burlingame, CA, USA). For negative controls, sections were processed in parallel under identical conditions with omission of anti-Fetuin-A alone, anti-Calbindin alone, or both primary antibodies. Images were acquired on an Olympus BX53F microscope (Olympus, Tokyo, Japan) at 200× and 400× magnifications, with identical exposure, gain and illumination settings across all groups. Fetuin-A expression in Purkinje cells was calculated as the proportion of Fetuin-A^+^/Calbindin^+^ double-positive cells relative to the Calbindin^+^/DAPI-positive Purkinje cell population and normalized to the Sham.

**TUNEL and Fluoro-Jade C.** TUNEL staining used a commercial kit (ab206386; Abcam, Cambridge, UK) according to the manufacturer’s protocol, the same kit used previously in this model [13]. DAB-positive nuclei were scored as apoptotic and quantified per unit length of the Purkinje cell layer. Fluoro-Jade C staining (TR-100-FJT; Biosensis, Thebarton, Australia) was combined with Calbindin immunofluorescence to identify degenerating Purkinje cells, quantified as the proportion of FJC^+^/Calbindin^+^ double-positive cells among Calbindin^+^/DAPI-positive cells and normalized to the Sham. TUNEL images are presented at 100× and 250× scanner magnifications, whereas FJC/Calbindin images were acquired using 200× and 400× magnifications. Where shown, inset images are digitally enlarged crops of the corresponding higher-magnification images and were not acquired at a separate magnification or used for quantification.

### 2.6. Primary Cerebellar Neuron Culture

Primary cerebellar cultures were prepared from E18 C57BL/6J embryos following the procedure established for cerebellar Fetuin-A studies in our laboratory [13]. Timed-pregnant dams were euthanized, embryos retrieved under sterile conditions, and cerebella dissected in ice-cold Hank’s Balanced Salt Solution (14185-052; Gibco, Grand Island, NY, USA) containing 10 µg/mL gentamicin (15710-064; Gibco, Grand Island, NY, USA). Tissue was rinsed in DMEM/F12 (21331020; Gibco, Grand Island, NY, USA), digested with 0.25% trypsin (from 2.5% stock, 15090046; Gibco, Grand Island, NY, USA) for 12 min at 37 °C, quenched with cold DMEM/F12, and triturated in 0.05% DNase I (11284932001; Roche, Basel, Switzerland).

Cells were resuspended in seeding medium (DMEM/F12 with 10% fetal bovine serum (A5669801; Gibco, Grand Island, NY, USA), 100 µM putrescine dihydrochloride (P5780; Sigma-Aldrich, St. Louis, MO, USA), 30 nM sodium selenite (214485; Sigma-Aldrich, St. Louis, MO, USA), 3.9 mM L-glutamine (A2916801; Gibco, Grand Island, NY, USA) and 3.5 µg/mL gentamicin) and plated at 1.2 × 10^6^ cells/mL in 96-well plates for MTT assays, 5 × 10^5^ cells/mL on coverslips coated with poly-L-ornithine (catalogue no. P4957; Sigma-Aldrich, St. Louis, MO, USA) for immunocytochemistry, 4.25 × 10^5^ cells/mL in 6-well plates for DCFDA and Annexin V/PI analyses, and 2.25 × 10^5^ cells/mL in 6-well plates for Western blotting. Four hours after plating, the medium was replaced with serum-free defined medium supplemented with 4 µM progesterone (P8783; Sigma-Aldrich, St. Louis, MO, USA), 20 µg/mL insulin (I6634; Millipore, Burlington, MA, USA) and 20 µg/mL transferrin (T8158; Sigma-Aldrich, St. Louis, MO, USA). Serum was deliberately withdrawn at this point, and all subsequent treatments and assays were performed under serum-free conditions, because fetal bovine serum contains abundant bovine fetuin and its retention would confound measurement of neuronal Fetuin-A. Cytosine β-D-arabinofuranoside (AraC; 4 µM; C1768; Sigma-Aldrich, St. Louis, MO, USA) was added at DIV 7 to suppress non-neuronal proliferation.

**Culture composition.** E18 dissociated cerebellar cultures are numerically dominated by granule-cell-lineage neurons, with Purkinje cells forming a minor subpopulation. Accordingly, all Purkinje-cell-resolved readouts (immunocytochemistry) were restricted to Calbindin-positive cells, whereas MTT, DCFDA, Annexin V/PI and Western blot data reflect the mixed cerebellar neuronal population and are interpreted as such. The same culture system supports robust Calbindin and Fetuin-A double labeling and was used to demonstrate the Fetuin-A dependence of cerebellar neuron survival [13].

### 2.7. Drug Treatments and In Vitro Experimental Design

Neurons were maintained until DIV 19. An MPP^+^ concentration–response experiment was performed at DIV 18 using 1-methyl-4-phenylpyridinium iodide (D048; Sigma-Aldrich, St. Louis, MO, USA) at 10–200 µM for 24 h; 10 µM was selected as the lowest concentration producing a reproducible, submaximal viability loss suitable for detecting protection (Supplementary Figure S3A). To identify effective antioxidant concentrations, cells were pretreated at DIV 17 for 24 h with RES, SDA or PIE before MPP^+^ challenge. Stocks of RES, SDA, and PIE were prepared in dimethyl sulfoxide (DMSO; SM-DIM1-050; GeneAll Biotechnology, Seoul, Republic of Korea) at 200 mg/mL and subsequently diluted in culture medium to the indicated concentrations. The final DMSO concentration, therefore, varied according to the treatment concentration, reaching a maximum of 0.5% (*v*/*v*) at the highest concentration tested during screening. At the working concentrations used in the main experiments, the final DMSO concentrations were 0.1% (*v*/*v*) for RES at 200 µg/mL and 0.0125% (*v*/*v*) for SDA and PIE at 25 µg/mL. To determine whether the maximum solvent concentration affected cell viability, an additional vehicle-control group containing 0.5% (*v*/*v*) DMSO alone was included in the MTT assay. MTT viability in this vehicle-control group did not differ significantly from that of the untreated control (*p* = 0.0742). Screening covered RES at 12.5–500 µg/mL and SDA and PIE at 25–1000 µg/mL. Working concentrations were selected based on MTT viability together with Fetuin-A immunocytochemistry: RES, 200 µg/mL; SDA, 25 µg/mL; and PIE, 25 µg/mL (Supplementary Figures S3–S6). Because Fetuin-A was the primary endpoint of this study, the concentration producing maximal recovery of Fetuin-A immunoreactivity was retained for each compound. For RES, this concentration did not coincide with the concentration producing the maximal MTT signal, and the consequences of this choice are addressed in Section 4.4.

### 2.8. Cell Viability, Immunocytochemistry, ROS and Cell-Death Assays

**MTT assay.** After treatment, cells in 96-well plates were incubated with MTT (5 mg/mL; M5655; Sigma-Aldrich, St. Louis, MO, USA) for 4 h at 37 °C; formazan was solubilized in DMSO and absorbance read at 570 nm on a Multiskan™ FC Photometer (Thermo Fisher Scientific, Waltham, MA, USA). Values were normalized to the untreated control. Because tetrazolium-based assays are susceptible to interference by reducing phytochemicals [50], the MTT data are interpreted alongside the independent Annexin V/PI and DCFDA readouts rather than in isolation.

**Immunocytochemistry.** Cells on coverslips were fixed in methanol at −20 °C, permeabilized with 0.2% Triton X-100 (SM-TRT-500; GeneAll Biotechnology, Seoul, Republic of Korea) and blocked with 5% bovine serum albumin (SM-BOV-100; GeneAll Biotechnology, Seoul, Republic of Korea), then incubated with anti-Fetuin-A (SC-133146; 1:100; Santa Cruz Biotechnology, Dallas, TX, USA) and anti-Calbindin D-28k (CB-38a; 1:500; Swant, Burgdorf, Switzerland), followed by Alexa Fluor 488- and 594-conjugated secondary antibodies (A21202 and A21207; Invitrogen, Carlsbad, CA, USA) and DAPI counterstaining. Images were acquired on an Olympus BX53F microscope (Olympus, Tokyo, Japan) with identical acquisition settings across groups. Fetuin-A expression was quantified as the proportion of Fetuin-A^+^/Calbindin^+^ double-positive cells among Calbindin^+^/DAPI-positive cells.

**ROS assay.** Intracellular ROS was quantified with the DCFDA/H2DCFDA Cellular ROS Assay Kit (ab113851; Abcam, Cambridge, UK). Cells were loaded with 20 µM DCFDA for 30 min at 37 °C in the dark and analyzed on a Cytek^®^ Guava^®^ easyCyte™ flow cytometer (Cytek Biosciences, Fremont, CA, USA). The mean fluorescence intensity (MFI) of the gated population was used for group comparisons.

**Cell-death assay.** Cell-death populations were assessed with the Tali™ Apoptosis Kit (Annexin V Alexa Fluor™ 488 and propidium iodide; A10788; Thermo Fisher Scientific). Cells were harvested, resuspended in Annexin-binding buffer and stained according to the manufacturer’s instructions, and then analyzed on the Cytek^®^ Guava^®^ easyCyte™ flow cytometer (Cytek Biosciences, Fremont, CA, USA). Cells were classified as viable (Annexin V^−^/PI^−^), early apoptotic (Annexin V^+^/PI^−^), late apoptotic (Annexin V^+^/PI^+^) or necrotic (Annexin V^−^/PI^+^). This quadrant-based classification describes the Annexin V/PI staining phenotype at the time of analysis and does not, by itself, distinguish among specific non-apoptotic cell-death mechanisms. Quadrant gates were set with unstained and single-stain controls prepared for each experiment, and harvesting conditions were kept identical across all groups within an experiment so that comparisons are made on a strictly relative basis.

### 2.9. Western Blot Analysis

Cerebellar tissue and primary cultured neurons were lysed in RIPA buffer (R0278; Sigma-Aldrich, St. Louis, MO, USA) supplemented with phosphatase inhibitor cocktail (P3200; GenDEPOT, Katy, TX, USA), as in our previous cerebellar Fetuin-A analyses [13]. Protein concentrations were determined with a BCA kit (21071; iNtRON Biotechnology, Seongnam, Republic of Korea). Equal amounts of protein were separated on 10% Tris-glycine gels and transferred to Amersham™ Hybond^®^ P PVDF membranes (GE10600023; Cytiva, Marlborough, MA, USA). After transfer, each membrane was cut horizontally between the 56 and 43 kDa molecular-weight marker positions, and the upper and lower membrane sections were processed separately for Fetuin-A and GAPDH, respectively, so that both signals were obtained from the same transferred membrane. Membranes were blocked with 5% BSA in TBST (TR2007-100-74; Biosesang, Yongin, Republic of Korea) and incubated with anti-Fetuin-A (SC-133146; mouse monoclonal; 1:100; Santa Cruz Biotechnology, Dallas, TX, USA) for 48 h at 4 °C or anti-GAPDH (#2118; rabbit monoclonal; 1:10,000; Cell Signaling Technology, Danvers, MA, USA) for 24 h at 4 °C, followed by species-matched HRP-conjugated secondary antibodies: anti-mouse IgG HRP-linked antibody (#7076; 1:1000; Cell Signaling Technology, Danvers, MA, USA) for Fetuin-A and goat anti-rabbit IgG (H+L) peroxidase (PI-1000; 1:2000; Vector Laboratories, Burlingame, CA, USA) for GAPDH. Signals were developed with a BS ECL Plus Kit (WC6002-200-KT; Biosesang, Yongin, Republic of Korea) and captured on a chemiluminescence bioimaging system (CELLGENTEK, Daejeon, Republic of Korea) with the exposure times chosen to avoid saturation. Band intensities were quantified in ImageJ (version 1.53; National Institutes of Health, Bethesda, MD, USA), and the Fetuin-A signals were normalized to GAPDH from the corresponding lower section of the same membrane. The corresponding uncropped Western blot images are provided in Supplementary Figure S7. Three technical limitations of this procedure should be noted. Marker bands were not documented on the lower membrane section, so the molecular weight of the GAPDH signal cannot be confirmed retrospectively. The membrane was cut close to the lower boundary of the Fetuin-A band, which carries a risk that part of that band was clipped. Samples were processed across separate gel strips, so normalization between strips may introduce variability that cannot be corrected after acquisition. A further immunoblot was subsequently performed on an intact, uncut membrane with the molecular-weight marker lane retained on the same image, and it is provided in Supplementary Figure S8. That blot was run on archived lysates as a separate experiment and was not used for quantification.

### 2.10. Statistical Analysis

Data are presented as the mean ± standard error of the mean (SEM). Analyses were performed in GraphPad Prism 10.5 (GraphPad Software, San Diego, CA, USA). Body weight, food intake, and water intake were analyzed by two-way repeated-measures ANOVA, with group as the between-subjects factor and time as the within-subjects factor, using the Geisser–Greenhouse correction and Tukey’s multiple-comparisons test. All other endpoints were analyzed by one-way ANOVA followed by Tukey’s multiple-comparisons test, with the family-wise alpha set at 0.05. For in vivo experiments, the experimental unit was the individual animal, and for in vitro experiments, *n* denotes the number of independent culture preparations rather than technical replicates. Image-based quantification was performed in ImageJ (version 1.53; National Institutes of Health, Bethesda, MD, USA) on coded files by an investigator blinded to group identity, whereas the DCFDA and Annexin V/PI flow-cytometry data were acquired and gated without blinding. Sample sizes for individual endpoints are provided in the figure legends.

## 3. Results

### 3.1. Antioxidant Treatments Attenuate Nigral Dopaminergic Loss and Improve Motor Outcomes

**Figure 1:**
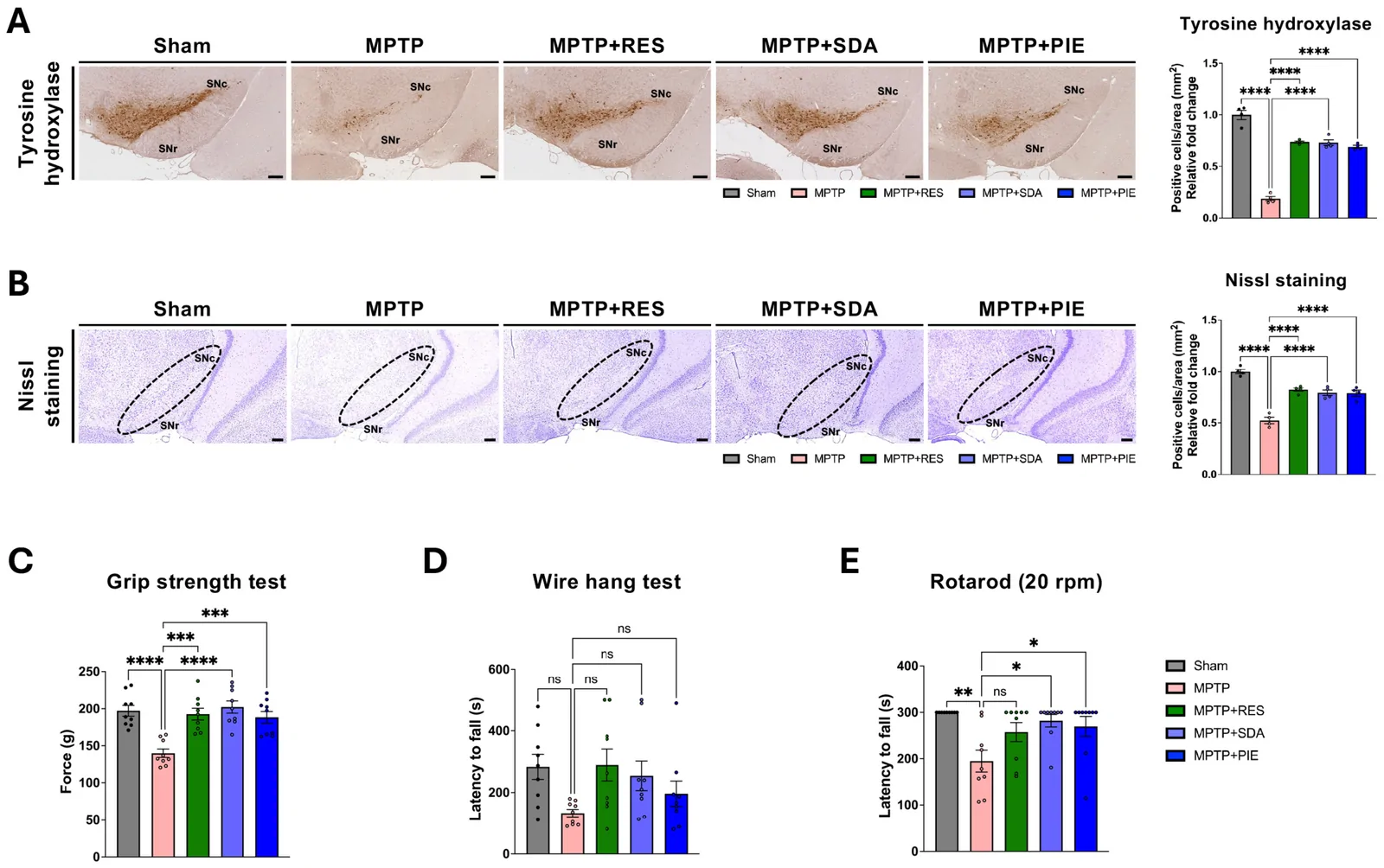
Antioxidant treatments attenuate nigral dopaminergic loss and improve motor outcomes in chronic MPTP-treated mice. (**A**) Representative tyrosine hydroxylase (TH) immunohistochemistry in the substantia nigra; TH-positive cells in the substantia nigra pars compacta (SNc) expressed as the fold change relative to the Sham (*n* = 4 mice/group). TH images are presented at a 6× scanner magnification. (**B**) Representative Nissl staining in the substantia nigra; Nissl-positive cells in the SNc expressed as the fold change relative to the Sham (*n* = 4 mice/group). Nissl images are presented at a 4× scanner magnification. The SNc and substantia nigra pars reticulata (SNr) are indicated in the representative images, and dashed ovals indicate the SNc region used for Nissl quantification. (**C**) Grip strength (g). (**D**) Wire-hang latency to fall (s). (**E**) Rotarod latency to fall at 20 rpm (s). Behavioral analyses used *n* = 9 mice/group. Scale bars: 100 µm. Data are presented as the mean ± SEM; one-way ANOVA with Tukey’s post hoc test. ns, not significant; * *p* < 0.05; ** *p* < 0.01; *** *p* < 0.001; and **** *p* < 0.0001 versus MPTP. The animals analyzed here are entirely separate from those studied in our parallel report [25]; see Section 2.2. SNc, substantia nigra pars compacta; SNr, substantia nigra pars reticulata.

MPTP exposure markedly reduced TH immunoreactivity in the SNc relative to the Sham, confirming that the dopaminergic lesion was established. RES, SDA and PIE each significantly increased the TH-positive signal relative to the MPTP group (Figure 1A). In none of the treatment groups did TH immunoreactivity return to the Sham levels, so the protection afforded was partial rather than complete. An additional Nissl analysis of the substantia nigra showed a concordant reduction after MPTP and preservation in the treated groups (Figure 1B). Motor testing showed a pronounced reduction in grip strength after MPTP that was significantly improved by all three treatments (Figure 1C). Wire-hang latency was reduced in the MPTP group, but differences between the MPTP and treatment groups did not reach significance (Figure 1D), consistent with the larger within-group variance of this assay. In the rotarod test, SDA and PIE significantly increased latency to fall relative to MPTP, with RES showing an intermediate response (Figure 1E). Body weight, food intake and water intake showed significant effects of time and group, with significant time × group interactions during the experimental period (Supplementary Figure S2). Despite these overall differences, the antioxidant-treated groups did not show sustained reductions in body weight, food intake or water intake over the experimental period. Together with the absence of mortality or treatment-related exclusion, these findings do not suggest that the behavioral effects of RES, SDA or PIE were attributable to generalized reductions in intake or overt systemic intolerance. Because *P. incarnata* preparations have documented sedative activity at higher doses [38,39], it is relevant that the PIE group did not show reduced rotarod latency or grip force relative to MPTP. The direction of the behavioral effect, thus, runs counter to a sedative confound at 50 mg/kg/day, although no dedicated test of sedation or arousal was performed. These nigral and behavioral findings reproduce, in an independent group of mice, those reported for this model in our parallel study [25] and are presented here to document that the model was established in the animals used for the cerebellar analyses that follow.

### 3.2. SDA and PIE Preserve Cerebellar Purkinje Cells and Cerebellar Fetuin-A

**Figure 2:**
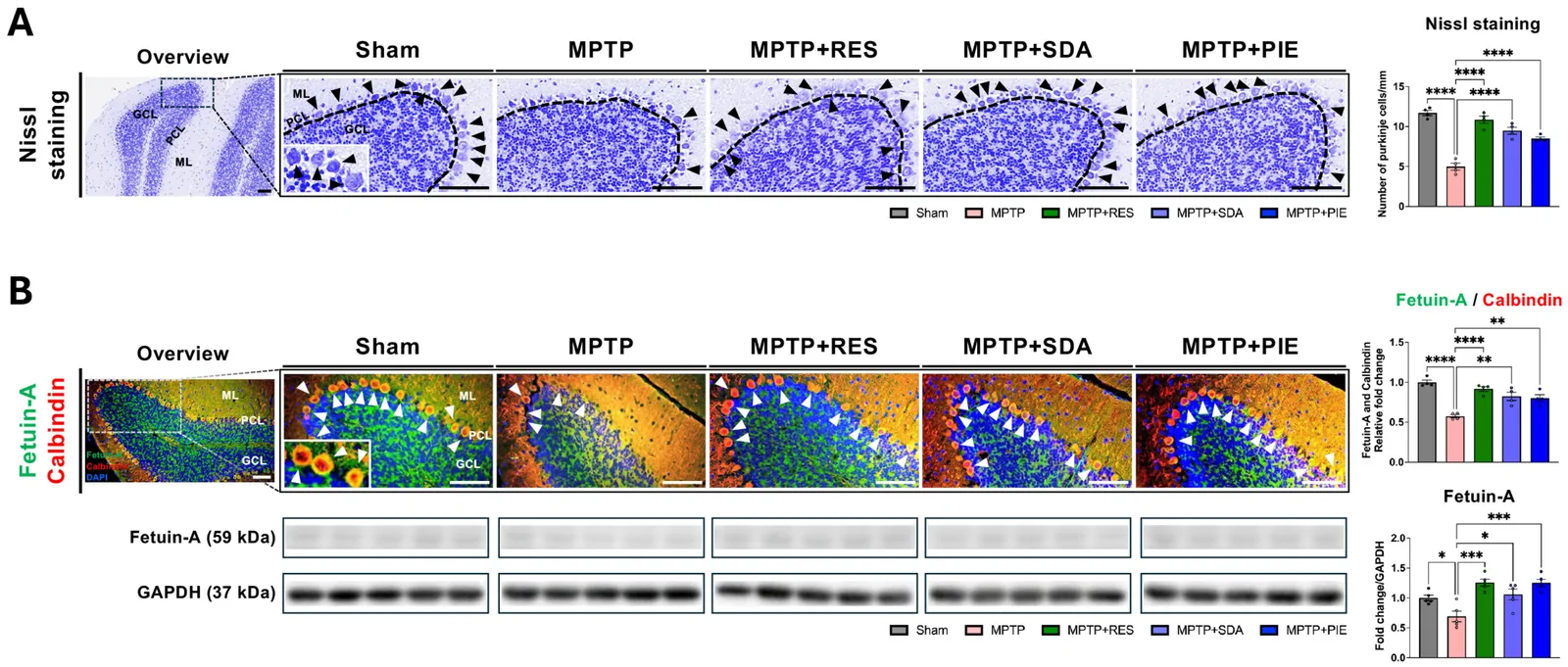
SDA and PIE preserve cerebellar Purkinje cells and cerebellar Fetuin-A in MPTP-treated mice. (**A**) Representative Nissl staining of the cerebellar cortex; Purkinje cell number expressed per millimeter (mm) of Purkinje cell layer (*n* = 4 mice/group). The overview image is presented at a 100× scanner magnification and the representative group images at a 400× scanner magnification. Arrowheads indicate the representative Nissl-positive Purkinje cells. (**B**) Representative immunofluorescence for Fetuin-A (green), Calbindin (red), and DAPI (blue), with yellow indicating colocalization of Fetuin-A and Calbindin; Fetuin-A expression in Calbindin-positive Purkinje cells is expressed as the fold change, relative to the Sham, of the fraction of Fetuin-A^+^/Calbindin^+^ double-positive cells among Calbindin^+^/DAPI-positive cells (*n* = 4 mice/group), together with representative Western blots of Fetuin-A (59 kDa) and GAPDH (37 kDa) in cerebellar tissue with densitometric quantification normalized to GAPDH (*n* = 5 mice/group). Immunofluorescence images were acquired at 200× and 400× magnifications. Arrowheads indicate representative Fetuin-A^+^/Calbindin^+^ double-positive Purkinje cells. Boxed regions in the overview images indicate the areas shown at higher magnification. Insets are digitally enlarged crops of the corresponding higher-magnification images and were not acquired at a separate magnification. Scale bars: 100 µm. Data are presented as the mean ± SEM; one-way ANOVA with Tukey’s post hoc test. * *p* < 0.05; ** *p* < 0.01; *** *p* < 0.001; and **** *p* < 0.0001 versus MPTP. GCL, granule cell layer; ML, molecular layer; PCL, Purkinje cell layer.

Nissl staining revealed a marked reduction in Purkinje cell number per millimeter (mm) of Purkinje cell layer after MPTP, reproducing the histological phenotype we previously documented in both MPTP-treated mice and human PD cerebella [13]. RES, SDA and PIE each significantly preserved Purkinje cell counts compared with MPTP alone (Figure 2A).

Double immunofluorescence showed reduced Fetuin-A signal in Calbindin-positive Purkinje cells in the MPTP group, again matching our earlier observation in this model and in patient tissue [13]. RES, SDA and PIE increased relative Fetuin-A expression compared with MPTP, while Western blotting of cerebellar tissue showed the same pattern: MPTP reduced Fetuin-A protein abundance, whereas antioxidant treatment restored it (Figure 2B). Because the immunoblot was performed on whole cerebellar lysate, it reports total cerebellar Fetuin-A rather than the Purkinje-cell-restricted pool. The concordance between the tissue-level and cell-resolved measurements indicates that the biochemical change is at least partly attributable to Purkinje cells. Importantly, the immunofluorescence readout is expressed as the proportion of Calbindin-positive Purkinje cells that are also Fetuin-A-positive; therefore, the reduction it reports is a change in the fraction of surviving Purkinje cells retaining Fetuin-A and not simply a consequence of the smaller number of Purkinje cells present after MPTP. To our knowledge, this is the first demonstration that the cerebellar Fetuin-A deficit associated with PD is pharmacologically reversible.

### 3.3. SDA and PIE Reduce Apoptotic and Degenerative Signals in Cerebellar Purkinje Cells

**Figure 3:**
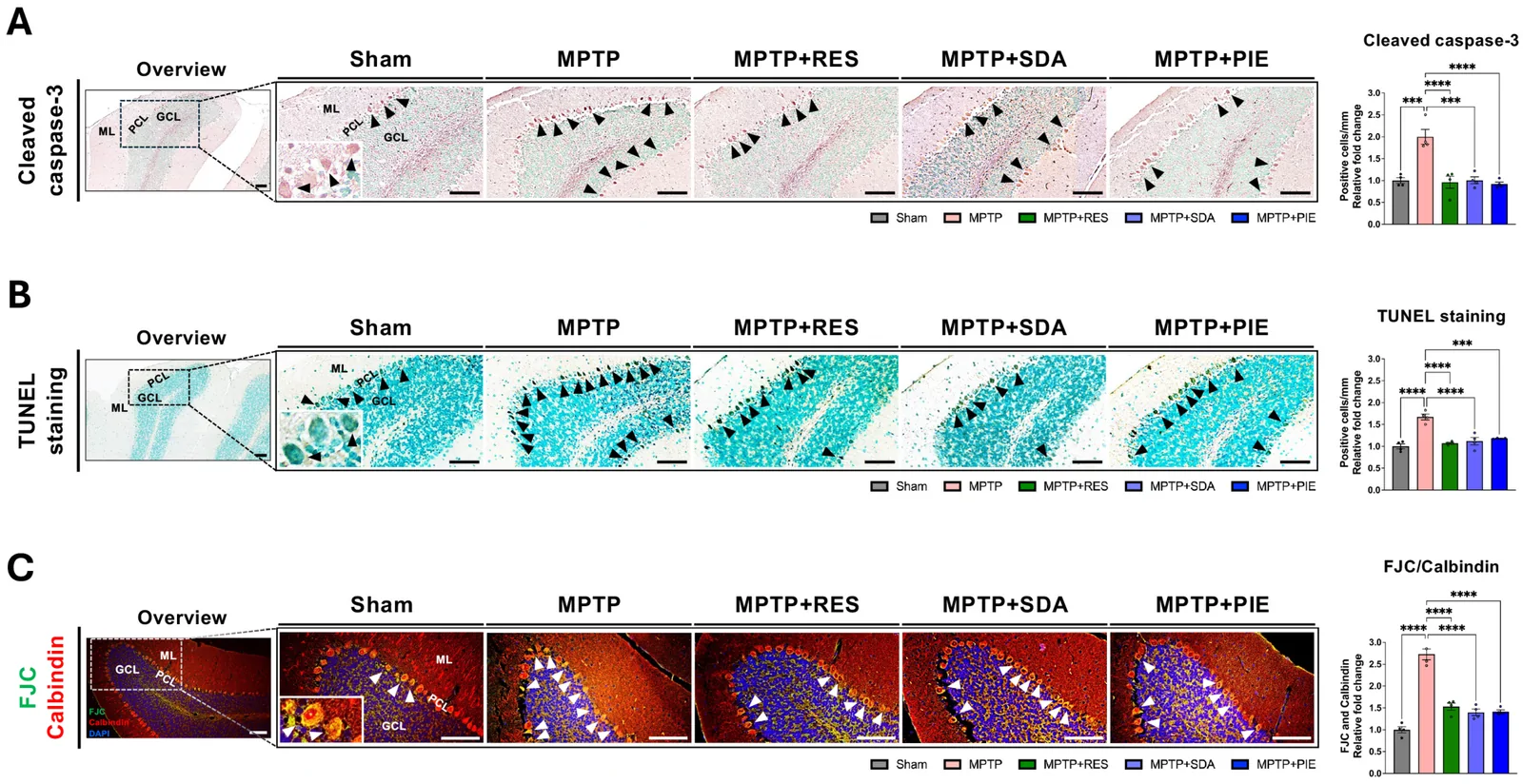
SDA and PIE attenuate apoptotic and degenerative signals in cerebellar Purkinje cells. (**A**) Representative cleaved caspase-3 (CC3) immunohistochemistry; CC3-positive cells in the Purkinje cell layer expressed as the fold change relative to the Sham. The overview image is presented at a 100× scanner magnification and the representative group images at a 250× scanner magnification. Arrowheads indicate representative CC3-positive cells in the Purkinje cell layer. (**B**) Representative TUNEL staining; TUNEL-positive cells are expressed as the fold change relative to the Sham. The overview image is presented at a 100× scanner magnification and the representative group images at a 250× scanner magnification. Arrowheads indicate representative TUNEL-positive cells in the Purkinje cell layer. (**C**) Representative Fluoro-Jade C (FJC; green), Calbindin (red) and DAPI (blue) staining, with yellow indicating colocalization of FJC and Calbindin; the fraction of FJC^+^/Calbindin^+^ double-positive cells among Calbindin^+^/DAPI-positive cells is expressed as the fold change relative to the Sham. FJC/Calbindin images were acquired at 200× and 400× magnifications. Arrowheads indicate representative FJC^+^/Calbindin^+^ double-positive Purkinje cells. Boxed regions in the overview images indicate the areas shown at higher magnification. Insets are digitally enlarged crops of the corresponding higher-magnification images and were not acquired at a separate magnification. Scale bars: 100 µm. Data are presented as the mean ± SEM (*n* = 4/group); one-way ANOVA with Tukey’s post hoc test. *** *p* < 0.001 and **** *p* < 0.0001 versus MPTP. GCL, granule cell layer; ML, molecular layer; PCL, Purkinje cell layer.

Cleaved caspase-3 immunohistochemistry showed a robust increase in positive cells in the Purkinje cell layer after MPTP, and RES, SDA and PIE each markedly reduced CC3 positivity (Figure 3A). TUNEL-positive cells were similarly increased by MPTP and reduced in the treated groups (Figure 3B). Fluoro-Jade C staining demonstrated an increased neurodegenerative signal associated with Calbindin-positive Purkinje cells after MPTP, which all three treatments significantly reduced (Figure 3C). Because cleaved caspase-3, TUNEL and FJC report mechanistically distinct features of neuronal injury, namely, executioner caspase activation, inter-nucleosomal DNA fragmentation and generalized neurodegeneration, their concordance supports genuine cerebellar vulnerability rather than an artifact of any single marker.

### 3.4. SDA and PIE Preserve Viability and Fetuin-A in MPP^+^-Challenged Primary Cerebellar Neurons

**Figure 4:**
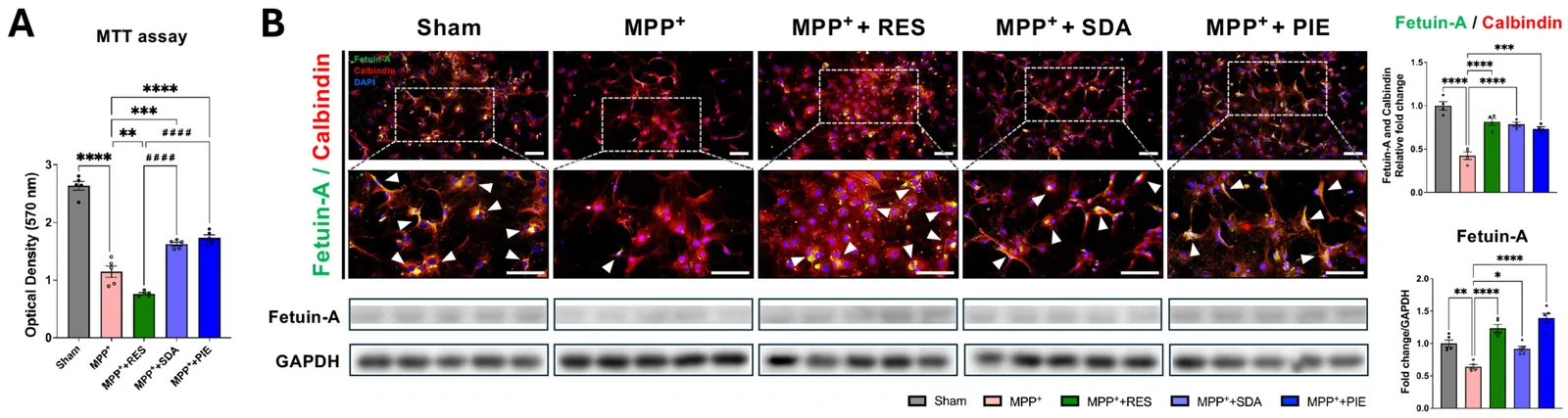
SDA and PIE preserve viability and Fetuin-A expression in MPP^+^-challenged primary cerebellar neurons. (**A**) MTT assay of cell viability at 570 nm (*n* = 5 independent culture preparations/group). (**B**) Representative immunocytochemistry for Fetuin-A (green), Calbindin (red), and DAPI (blue), with yellow indicating colocalization of Fetuin-A and Calbindin; the Fetuin-A signal in Calbindin-positive cells is expressed as the fold change, relative to the Sham, of the fraction of Fetuin-A^+^/Calbindin^+^ double-positive cells (*n* = 4 independent culture preparations/group). Immunocytochemistry images were acquired at 200× and 400× magnifications. Arrowheads indicate representative Fetuin-A^+^/Calbindin^+^ double-positive cells. Representative Western blots of Fetuin-A (59 kDa) and GAPDH (37 kDa) with densitometric quantification normalized to GAPDH (*n* = 5 independent culture preparations/group). RES was applied at 200 µg/mL and SDA and PIE at 25 µg/mL. All treatments were performed in serum-free defined medium to exclude serum-derived fetuin. Scale bars: 100 µm. Data are presented as the mean ± SEM; one-way ANOVA with Tukey’s post hoc test. * *p* < 0.05; ** *p* < 0.01; *** *p* < 0.001; and **** *p* < 0.0001 versus MPP^+^; ^####^ *p* < 0.0001 versus MPP^+^+RES. Sham, untreated cells.

MPP^+^ at 10 µM significantly reduced the MTT signal relative to untreated cells. At the concentrations selected for the main experiment, SDA and PIE, both at 25 µg/mL, significantly increased cell viability relative to MPP^+^ alone, whereas RES at 200 µg/mL did not rescue the MTT signal and yielded values significantly lower than those of SDA and PIE (Figure 4A). Section 4.4 addresses this behavior of RES.

Despite these differences in the MTT signal, Fetuin-A was consistently responsive to antioxidant treatment. MPP^+^ reduced Fetuin-A abundance by Western blotting and decreased the fraction of Fetuin-A-positive Calbindin-positive cells by immunocytochemistry, whereas RES, SDA and PIE partially restored both measures (Figure 4B). Because all in vitro treatments were performed in serum-free defined medium, the Fetuin-A measured here reflects neuronal rather than serum-derived protein. Concentration–response experiments supported the selection of the MPP^+^ challenge and documented the non-monotonic effects of the three compounds on viability and Fetuin-A expression (Supplementary Figures S3–S6).

### 3.5. All Three Antioxidants Suppress MPP^+^-Induced DCFDA Signal, and MPP^+^ Increases the Annexin V^−^/PI^+^ Necrotic Population in Culture

**Figure 5:**
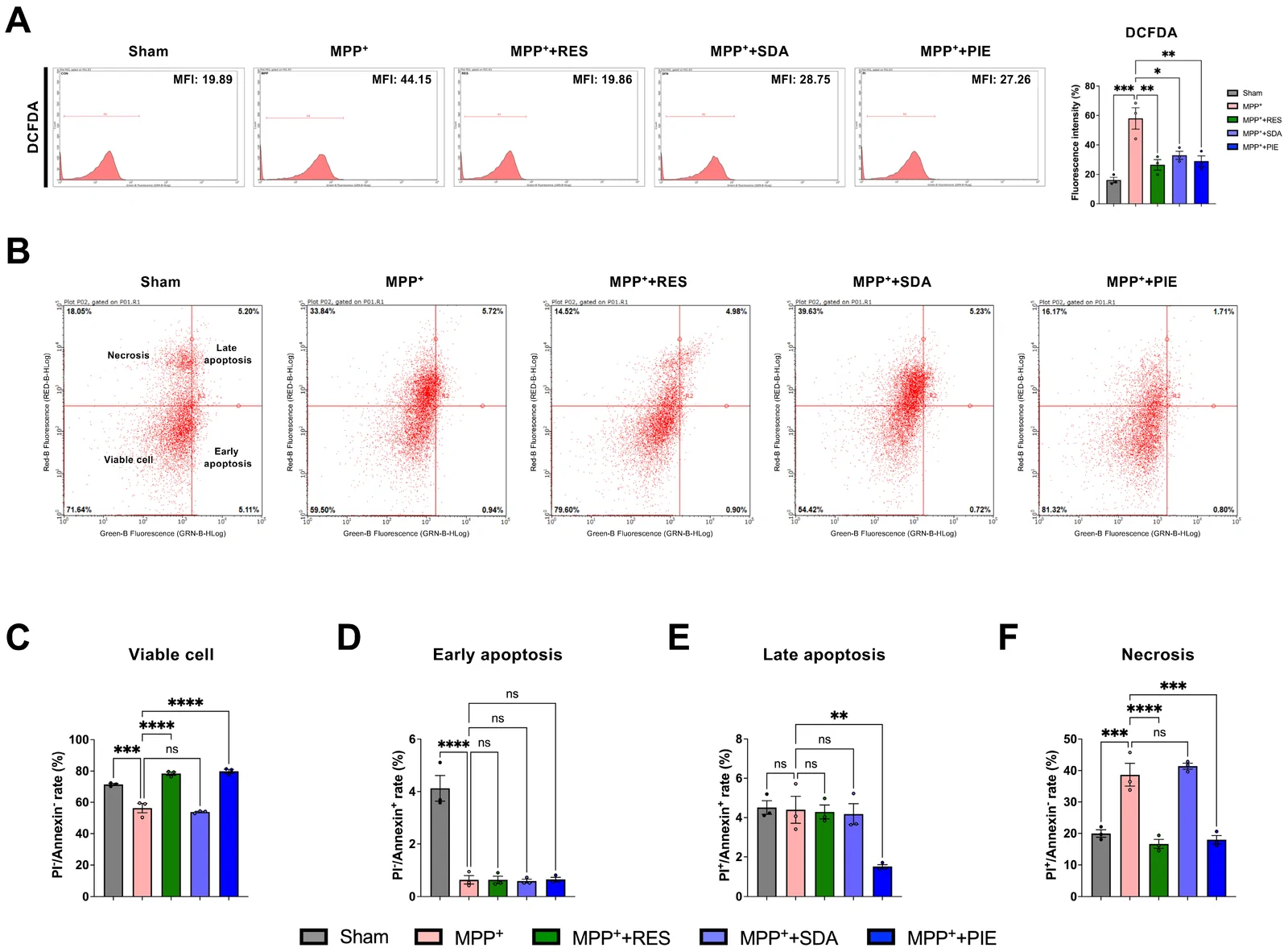
SDA and PIE reduce the MPP^+^-induced DCFDA signal, and MPP^+^ increases the Annexin V−/PI+ necrotic population in mixed primary cerebellar cultures. (**A**) Representative DCFDA histograms; mean fluorescence intensity (MFI) values of the displayed experiment are annotated, and the right panel shows the quantified DCFDA fluorescence across independent culture preparations. (**B**) Representative Annexin V Alexa Fluor 488 and propidium iodide (PI) dot plots; quadrant percentages are those of the single representative experiment shown. (**C**–**F**) Quantification across independent culture preparations of (**C**) viable cells (Annexin V^−^/PI^−^), (**D**) early apoptotic cells (Annexin V^+^/PI^−^), (**E**) late apoptotic cells (Annexin V^+^/PI^+^) and (**F**) necrotic cells (Annexin V^−^/PI^+^). Data are presented as the mean ± SEM (*n* = 3 independent culture preparations/group), the analysis unit is the independent culture preparation, and comparisons used one-way ANOVA with Tukey’s post hoc test. ns, not significant; * *p* < 0.05; ** *p* < 0.01; *** *p* < 0.001; and **** *p* < 0.0001 versus MPP^+^. In panels (**B**–**F**) and in the quadrant designations, PI has its conventional flow-cytometric meaning of propidium iodide; elsewhere in this paper, PIE denotes the *Passiflora incarnata* extract (see Section 2.1).

DCFDA analysis showed that MPP^+^ increased intracellular ROS. In the representative experiment shown in Figure 5A, MFI rose from 19.89 in untreated cells to 44.15 after MPP^+^ and was reduced to 19.86, 28.75, and 27.26 by RES, SDA, and PIE, respectively. Across independent preparations, MPP+ significantly increased the quantified fluorescence intensity, whereas all three interventions significantly reduced it (Figure 5A, *n* = 3 independent preparations per group). All three treatments, therefore, suppressed MPP^+^-associated ROS accumulation in cerebellar neurons.

Annexin V Alexa Fluor 488/propidium iodide profiling revealed a distinctive death signature (Figure 5B–F). The viable fraction was reduced by MPP^+^ and significantly increased by RES and PIE, whereas SDA did not improve this parameter at 25 µg/mL (Figure 5C). The Annexin V^−^/PI^+^ fraction was increased by MPP^+^ and significantly reduced by RES and PIE (Figure 5F), and PIE additionally reduced the late-apoptotic fraction (Figure 5E). Importantly, the early-apoptotic (Annexin V^+^/PI^−^) fraction was lower, not higher, in all MPP^+^-exposed conditions than in untreated cultures (Figure 5D), whereas the Annexin V^−^/PI^+^ necrotic population was markedly increased after MPP^+^ exposure. At the single 24 h sampling point, this pattern indicates that phosphatidylserine-externalizing early apoptosis was not the dominant flow-cytometric signature of MPP^+^-induced cell death. However, Annexin V/PI profiling alone cannot distinguish among specific non-apoptotic death mechanisms or exclude apoptotic cells that had progressed to secondary membrane permeabilization. This pattern is consistent with our earlier finding that Fetuin-A silencing in the same culture system reduced the Calbindin-positive cell number without producing TUNEL positivity [13]. In contrast, the concurrent increases in cleaved caspase-3 and TUNEL in the cerebellum of MPTP-treated mice demonstrate that an apoptotic component was also present in vivo. Thus, the in vitro and in vivo findings indicate coexisting cell-death responses rather than an exclusively non-apoptotic process. Group values are reported as the mean ± SEM in Figure 5C–F; the percentages annotated on the dot plots in Figure 5B are those of the single representative experiment displayed.

## 4. Discussion

The present study extends our previous observation of reduced cerebellar Fetuin-A in human PD and in MPTP-treated mice [13] by showing that this reduction is pharmacologically reversible in this model. The principal findings are that (i) chronic MPTP produced nigral dopaminergic injury and motor impairment together with cerebellar Purkinje cell loss, (ii) cerebellar injury was accompanied by reduced Fetuin-A and increased apoptotic and degenerative markers, (iii) SDA and PIE preserved Purkinje cell integrity and restored Fetuin-A, and (iv) in primary cerebellar neurons, MPP^+^ increased ROS and reduced Fetuin-A, while all three interventions lowered ROS and restored Fetuin-A.

### 4.1. Convergent Redox Evidence for SDA and PIE in This Model

The antioxidant framing of these results does not rest on the in vitro DCFDA data alone. In our parallel analysis of an independent group of mice treated with the repeated MPTP dosing regimen, MPTP increased the astrocytic NOX4–MPO–OPN axis and the lipid-peroxidation adduct 4-HNE and reduced mitochondrial import proteins TIM23 and TOM20, along with all five oxidative phosphorylation complexes. Oral SDA and PIE at the same 50 mg/kg/day dose normalized each of these markers [25]. Because NOX4 is a sustained, constitutively active source of ROS whose principal downstream consequence is lipid peroxidation [20,21,22] and because astrocytic redox dysfunction propagates to neighboring neurons [51,52], those measurements provide supporting evidence that the two extracts influence redox-related processes in vivo in this model at the dose used here. However, they were obtained in a separate group of animals and in a different brain region, and we treat them as supporting evidence for the redox-related activity of these preparations rather than as a direct demonstration of cerebellar redox modulation in the animals analyzed here. The present cerebellar data should be read against that background. The MPP^+^-induced rise in DCFDA fluorescence and its suppression by SDA and PIE are consistent with the tissue-level redox changes documented for this model, but DCFDA does not identify the source of intracellular oxidants, the extent of lipid peroxidation, or the status of endogenous antioxidant pathways.

### 4.2. Cerebellar Purkinje Cells as a Site of PD-Associated Redox Injury

PD is increasingly understood as a network disorder extending beyond the nigrostriatal pathway [7,8,9]. MPTP has long been known to alter calcium-binding protein expression in primate Purkinje cells and to drive their persistent activation in parallel with nigral degeneration [10,11], and we previously showed that Purkinje cell number and morphology are abnormal in both MPTP-treated mice and human PD cerebella [13]. Here, MPTP reduced Purkinje cell density and increased cleaved caspase-3, TUNEL, and FJC signals, and all three antioxidants attenuated these changes. The intrinsic properties of Purkinje cells, namely, tonic high-frequency firing, large dendritic arbors, and heavy reliance on calcium handling, impose a sustained oxidative load that plausibly underlies this vulnerability [12]. The functional relevance of cerebellar preservation is compatible with the improvements in grip strength and rotarod performance, but the present design cannot attribute behavioral rescue specifically to cerebellar rather than nigrostriatal protection, since both compartments were protected in the same animals.

### 4.3. Fetuin-A as a Treatment-Responsive Correlate and the Cell-Death Profile

The treatment responsiveness of Fetuin-A is the central novel observation of this work. Fetuin-A has broad metabolic, inflammatory and cytoprotective roles [14,15,16,17,18], and after traumatic brain injury, it suppresses microglial necroptosis through NRF2/HO-1 signaling [19]. These observations, together with the present findings, suggest a possible relationship between Fetuin-A preservation and redox regulation; however, this study does not establish whether Fetuin-A lies upstream or downstream of redox signaling, and it did not directly assess NRF2/HO-1 pathway activation in cerebellar tissue. Notably, vitexin, the marker constituent of the PIE preparation used here, has itself been shown to augment NRF2/HO-1-dependent antioxidant signaling [41] and to protect dopaminergic neurons in MPTP-treated mice [40], providing additional context for a potential relationship between PIE and this pathway, although this mechanism was not directly examined in the present study.

The Annexin V/PI results are informative rather than anomalous in this context. The increment in cell death after MPP^+^ was associated with a marked increase in the Annexin V^−^/PI^+^ necrotic population, while the Annexin V^+^/PI^−^ population decreased, indicating that early phosphatidylserine-externalizing apoptosis was not the dominant flow-cytometric signature at the 24 h sampling point. This pattern does not, however, establish a specific non-apoptotic cell-death mechanism, and Annexin V/PI profiling cannot exclude cells that have progressed from apoptosis to secondary membrane permeabilization. This is compatible with our earlier loss-of-function experiment, in which Fetuin-A silencing in the same E18 cerebellar culture system reduced the Calbindin-positive cell number yet produced no increase in TUNEL positivity [13]. It is also compatible with, but does not directly demonstrate, the mechanism documented in our parallel study of this model, where NOX4-driven lipid peroxidation promotes a ferroptosis-like, non-apoptotic death that terminates in plasma-membrane permeabilization [20,21,25]. The difference from the in vivo cerebellar data, where cleaved caspase-3 and TUNEL both increased, indicates that apoptotic signaling is also present in intact tissue. The in vivo cerebellum contains a heterogeneous cell population, whereas the dissociated culture reflects a mixed neuronal population examined at a single time point. Taken together, the two data sets support the coexistence of apoptotic and non-apoptotic cell-death responses rather than identifying one pathway as predominant.

### 4.4. Compound-Specific Behavior and the Interpretation of the MTT Data

Two technical considerations bear directly on the in vitro comparisons. First, RES was used at 200 µg/mL, corresponding to approximately 0.9 mM, far above concentrations achievable in vivo. MTT and flow cytometry do not measure the same quantity. MTT reports NAD(P)H-dependent tetrazolium reduction, which tracks mitochondrial dehydrogenase activity summed across the well, whereas Annexin V/PI profiling reports the proportion of cells that retain plasma-membrane integrity without phosphatidylserine externalization. A compound can preserve membrane integrity while at the same time depressing mitochondrial reductive capacity, and the two readouts then diverge. Resveratrol binds and inhibits mitochondrial F_1_-ATPase at micromolar concentrations [53], so at approximately 0.9 mM, a flat MTT signal, together with improved Annexin V/PI distribution and reduced DCFDA fluorescence, is the combination predicted rather than a contradiction. We, therefore, do not claim a protective effect of RES by the MTT criterion, and the viability statement for RES rests on the flow-cytometric data. This concentration was retained only because it produced the maximal recovery of Fetuin-A immunoreactivity, which was the primary endpoint of the screen. Because an RES-only condition was not included, the present data cannot determine whether RES itself contributed to the reduced MTT signal at 200 µg/mL. Second, tetrazolium-based viability assays are known to be susceptible to interference from reducing compounds and to depend on mitochondrial dehydrogenase activity rather than on cell number as such [50]. For polyphenol- and flavonoid-rich preparations, MTT data are best read alongside orthogonal measures, which is why the viability conclusions here are anchored on concordance among MTT, Annexin V/PI, and Fetuin-A immunocytochemistry rather than on MTT alone.

SDA reduced ROS and restored Fetuin-A but did not improve the Annexin V/PI distribution at 25 µg/mL, whereas its in vivo protection was robust. A narrow in vitro efficacy window, the different timescales on which redox suppression and membrane integrity are measured, and the metabolic conversion of orally administered apocarotenoids into active species that are absent during direct neuronal exposure [26,27,28] are all plausible and non-exclusive explanations that remain to be tested.

### 4.5. Alternative Interpretations Considered

Three alternative readings of these data deserve explicit consideration. First, the preservation of Fetuin-A could be a passive consequence of Purkinje cell survival rather than a participant in it. Two observations argue against a purely passive relationship. The immunofluorescence measure is a per-cell proportion among surviving Calbindin-positive neurons rather than an absolute count, and in vitro, the loss of Fetuin-A was evident under a submaximal viability deficit. Nevertheless, the present design cannot exclude that Fetuin-A tracks cellular health generically, and only loss- and gain-of-function experiments can settle this. Second, the cerebellar effects could be secondary to nigrostriatal protection rather than a direct cerebellar action. The in vitro data, in which MPP+ reduced Fetuin-A and viability in isolated cerebellar neurons with no dopaminergic input, indicate that a cell-autonomous cerebellar effect exists, but the relative contribution of the two compartments in vivo remains undetermined. Third, because Fetuin-A is an abundant circulating liver-derived protein, cerebellar signal could, in principle, reflect plasma contamination or altered blood–brain barrier permeability rather than neuronal expression. The in vitro experiments were performed in serum-free defined medium and, therefore, report neuronal Fetuin-A independently of any serum source, and the in vivo tissue was obtained after transcardial saline perfusion. A circulating contribution to the tissue immunoblot cannot, however, be formally excluded, and paired measurement of serum and cerebellar Fetuin-A would resolve it.

### 4.6. Chemical Basis and Translational Considerations

The two preparations differ in phytochemical class, which may explain their partly divergent profiles. SDA is enriched in apocarotenoids such as crocin and crocetin, which have been associated with dopaminergic neuroprotection, mitochondrial protection and motor improvement in MPTP and related models [29,30,31,32,33], and standardized saffron extracts from this supplier have documented safety and efficacy in the clinical literature [35,36,37]. PIE contains flavone C-glycosides, principally vitexin together with isovitexin, orientin and isoorientin [38,39], a class repeatedly linked to antioxidant and neuroprotective activity [40,41,54,55,56,57,58,59]. Both are food-grade, standardized preparations with established human exposure, which is a practical advantage for any long-term nutritional strategy in a chronic neurodegenerative disease.

### 4.7. Limitations

Several limitations should be acknowledged. First, cell-free radical-scavenging assays (DPPH, ABTS, and FRAP) and batch-specific quantification of marker compounds were not performed on the extract lots used here. We relied instead on the published analytical standardization of these preparations and their marker constituents [34,38,39], on prior functional antioxidant characterization of the same PIE lineage in *C. elegans* [38], and on the in vivo redox measurements obtained in a separate group of mice in our parallel study [25]. No oxidative-damage or endogenous antioxidant-response marker was measured in the cerebellar tissue of the animals analyzed here, and the redox interpretation of the in vivo findings, therefore, rests on convergent rather than direct evidence. In addition, DCFDA measurements were limited to primary cerebellar cultures and provide a broad readout of intracellular oxidant-sensitive fluorescence rather than pathway-specific evidence. Measurement of 4-HNE, glutathione status, and NRF2/HO-1/NQO1 expression in cerebellar tissue is the immediate next step. Batch-resolved phytochemical and radical-scavenging profiling should accompany future work. Second, the present experiments establish Fetuin-A as a treatment-responsive correlate, not as a proven causal mediator. To test necessity and sufficiency, future work should include Fetuin-A knockdown, neutralization, or supplementation under SDA and PIE treatment, and direct measurement of NRF2, HO-1, and NQO1 in cerebellar tissue is the natural next step. Third, oral treatment began 1 week before MPTP and continued during toxin exposure, so the design evaluates a preventive paradigm rather than reversal of established disease. Fourth, a single dose level (50 mg/kg/day) was tested in vivo, precluding characterization of the dose–response relationship. Extract-only control groups were also not included, either in vivo or in vitro. Every comparison reported here is made against the toxin plus vehicle group, and the findings, therefore, describe attenuation of toxin-induced change. The baseline effects of SDA and PIE on the intact nigrostriatal and cerebellar systems and on Fetuin-A expression in the absence of MPTP/MPP^+^ exposure were not determined. Fifth, only male mice were studied, and sex-dependent differences in MPTP susceptibility are well documented. Sixth, sample sizes for histology (*n* = 4) and flow cytometry (*n* = 3) were modest. In addition, Annexin V/PI profiling was performed at a single 24 h time point and cannot distinguish necrosis from specific regulated non-apoptotic mechanisms or exclude apoptosis that has progressed to secondary membrane permeabilization; pathway-specific markers and inhibitor-rescue experiments will be required to define the underlying cell-death mechanism. Seventh, the immunoblot procedure carries three specific technical limitations, which are described in Section 2.9. Molecular-weight marker bands were not documented on the lower membrane section, so the molecular weight of the GAPDH signal cannot be confirmed retrospectively. The membrane was cut close to the lower boundary of the Fetuin-A band, which risks clipping part of that band. Samples were processed across separate gel strips, so normalization between strips may introduce variability that cannot be corrected after acquisition. These points should be considered when interpreting the Fetuin-A immunoblot data. A further immunoblot performed on an intact membrane, with the molecular-weight marker lane retained on the same image, is provided as Supplementary Figure S8. That blot was run on archived lysates as a separate experiment and was not quantified, so it documents the procedure without verifying the original membranes retrospectively.

Eighth, although the repeated-dosing MPTP regimen used here produces a sustained rather than an acute insult, it remains a toxin-induced model that does not reproduce the progressive, α-synuclein-associated pathology of idiopathic PD. Validation in progressive or genetic models will be necessary. Pharmacokinetic and brain-exposure measurements were not performed for SDA, PIE, or their constituent metabolites in the present study. The extent to which the administered preparations or their metabolites reached the cerebellum, therefore, remains undetermined.

Ninth, cerebellar Fetuin-A was not measured in parallel with circulating Fetuin-A, so the contribution of plasma-derived protein to the tissue immunoblot cannot be formally excluded. Tenth, multiple endpoints were compared across five groups without correction across endpoint families. We regard the analyses as estimating a consistent pattern of effect rather than as independent confirmatory tests, and the concordance among mechanistically distinct markers is the principal basis for our interpretation. Finally, the primary cerebellar cultures contain a mixed-neuronal population in which Purkinje cells are a minority, so the bulk biochemical readouts are not Purkinje-cell-specific.

## 5. Conclusions

SDA and *Passiflora incarnata* L. extract attenuated multiple features of chronic MPTP/MPP^+^-associated neurotoxicity, including increased intracellular DCFDA fluorescence, cerebellar Purkinje cell injury, and loss of Fetuin-A expression. The findings show, for the first time, that the cerebellar Fetuin-A deficit previously identified in human PD and in MPTP-treated mice is responsive to SDA and PIE treatment, while the cell-death data indicate the coexistence of apoptotic and non-apoptotic components rather than a single predominant death pathway. Together with the redox-inflammatory and mitochondrial changes documented for this model [25], these results identify cerebellar Fetuin-A as a treatment-responsive correlate of Purkinje cell integrity associated with reduced intracellular oxidant-sensitive fluorescence, while the specific antioxidant pathways involved remain to be established.

## Data Availability

The original contributions presented in this study are included in the article and its Supplementary Materials. Further inquiries can be directed to the corresponding author.

## Supplementary Materials

The following supporting information can be downloaded at: https://www.mdpi.com/article/10.3390/antiox15101319/s1, Figure S1: Experimental design and treatment schedule; Figure S2: Body weight, food intake and water intake; Figure S3: Determination of MPP^+^- and antioxidant-treatment concentration ranges; Figure S4: Concentration-dependent effects of RES on Fetuin-A expression; Figure S5: Concentration-dependent effects of SDA on Fetuin-A expression; Figure S6: Concentration-dependent effects of PIE on Fetuin-A expression; Figure S7: Original Western blot images of the Fetuin-A and GAPDH membrane sections; Figure S8: Fetuin-A and GAPDH detected on an intact membrane; Table S1: Animal allocation and use for each investigative technique.

## Abbreviations

The following abbreviations are used in this manuscript:

4-HNE	4-hydroxynonenal
*AHSG*	alpha-2-HS-glycoprotein (gene symbol for Fetuin-A)
ANOVA	analysis of variance
AraC	cytosine β-D-arabinofuranoside
ARRIVE	Animal Research: Reporting of In Vivo Experiments
BCA	bicinchoninic acid
BSA	bovine serum albumin
CC3	cleaved caspase-3
DAB	3,3′-diaminobenzidine
DAPI	4′,6-diamidino-2-phenylindole
DCFDA	2′,7′-dichlorofluorescin diacetate
DIV	days in vitro
DMEM/F12	Dulbecco’s Modified Eagle Medium/Nutrient Mixture F-12
DMSO	dimethyl sulfoxide
E18	embryonic day 18
FJC	Fluoro-Jade C
GAPDH	glyceraldehyde-3-phosphate dehydrogenase
GCL	granule cell layer
HO-1	heme oxygenase-1
HPLC	high-performance liquid chromatography
HRP	horseradish peroxidase
LC-MS/MS	liquid chromatography–tandem mass spectrometry
MFI	mean fluorescence intensity
ML	molecular layer
MPO	myeloperoxidase
MPP^+^	1-methyl-4-phenylpyridinium
MPTP	1-methyl-4-phenyl-1,2,3,6-tetrahydropyridine
MTT	3-(4,5-dimethylthiazol-2-yl)-2,5-diphenyltetrazolium bromide
NOX4	NADPH oxidase 4
NQO1	NAD(P)H quinone dehydrogenase 1
NRF2	nuclear factor erythroid 2-related factor 2
OPN	osteopontin
PCL	Purkinje cell layer
PD	Parkinson’s disease
PI	propidium iodide
PIE	*Passiflora incarnata* L. extract
RES	resveratrol
RIPA	radioimmunoprecipitation assay
ROS	reactive oxygen species
SDA	saffron-derived antioxidant (*Crocus sativus* L. extract)
SEM	standard error of the mean
SNc	substantia nigra pars compacta
SNr	substantia nigra pars reticulata
TBST	tris-buffered saline containing 0.1% Tween 20
TH	tyrosine hydroxylase
TIM23	translocase of the inner mitochondrial membrane 23
TOM20	translocase of the outer mitochondrial membrane 20
TUNEL	terminal deoxynucleotidyl transferase dUTP nick-end labeling
