# Supplementary material for: Saffron-Derived Antioxidant and Passiflora incarnata L. Extract Preserve Cerebellar Fetuin-A and Protect Purkinje Cells Against MPTP/MPP+-Induced Injury
Source: Antioxidants (Basel). 2026 Oct 13;15(10):1319. doi: 10.3390/antiox15101319 (PMC13701996; doi:10.3390/antiox15101319)
Supplement: Supplementary file 1 [file antioxidants-15-01319-s001.zip › antioxidants-4571606-supplementary.pdf]

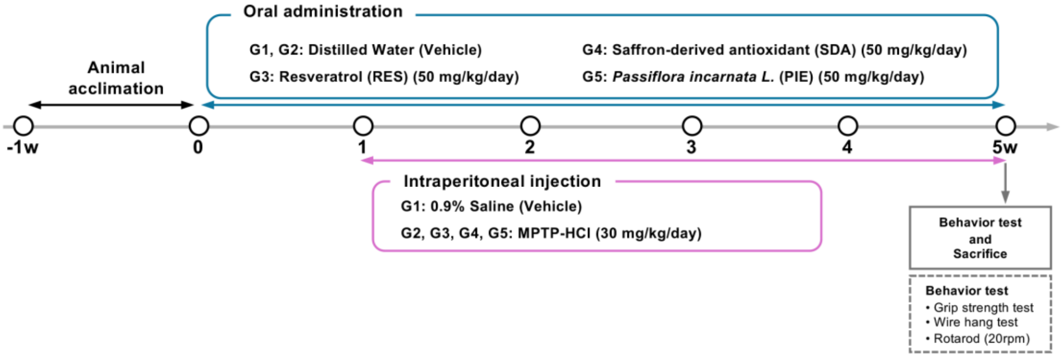

Figure S1. Experimental design and treatment schedule. Mice were acclimatized for 1 week and then assigned to five groups. Sham mice received distilled water orally and 0.9% saline intraperitoneally. MPTP mice received distilled water orally and MPTP-HCl (30 mg/kg/day, i.p.), whereas the treatment groups received RES, SDA or PIE orally at 50 mg/kg/day. Oral administration was continued for 5 weeks, and MPTP or saline injections were given during the final 4 weeks. At week 5, grip strength, wire-hang and rotarod tests were performed, followed by sacrifice. RES, resveratrol; SDA, saffron-derived antioxidant; PIE, *Passiflora incarnata* L. extract; MPTP, 1-methyl-4-phenyl-1,2,3,6-tetrahydropyridine.

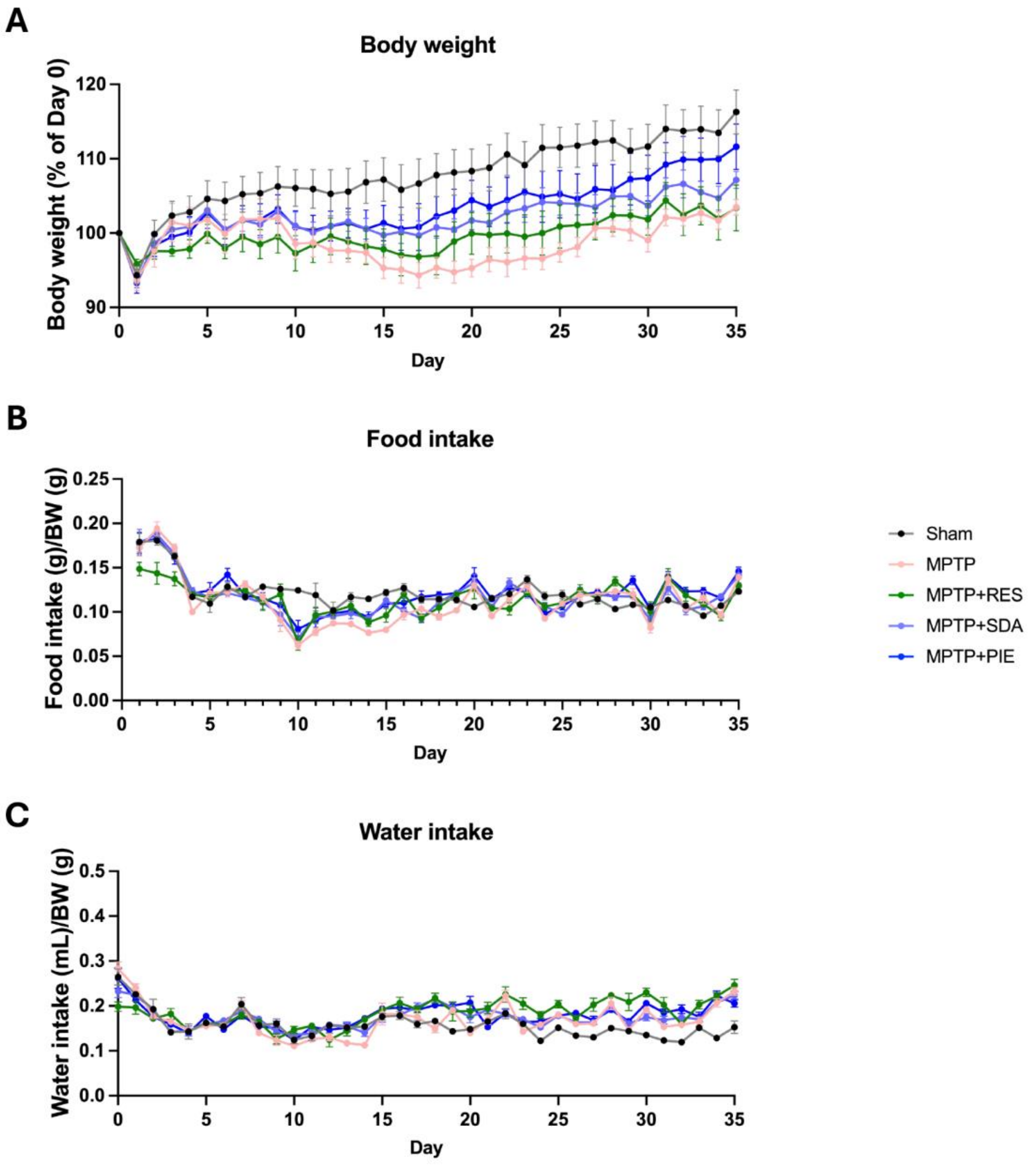

Figure S2. Body weight, food intake and water intake. (**A**) Changes in body weight during the experimental period, expressed as a percentage of the Day 0 value. (**B**) Daily food intake normalized to body weight. (**C**) Daily water intake normalized to body weight. Data are mean ± SEM (n = 9 mice/group) and were analyzed by two-way repeated-measures ANOVA with Geisser–Greenhouse correction, followed by Tukey's multiple-comparisons test. For body weight, significant effects of time ($F(3.399, 135.9) = 25.58$, $p < 0.0001$) and group ($F(4, 40) = 3.578$, $p = 0.0138$), as well as a significant time × group interaction ($F(13.59, 135.9) = 2.251$, $p = 0.0094$), were observed. For food intake, significant effects of time ($F(2.358, 94.31) = 60.08$, $p < 0.0001$) and group ($F(4, 40) = 4.190$, $p = 0.0063$), with a significant time × group interaction ($F(9.431, 94.31) = 3.740$, $p = 0.0004$), were observed. For water intake, significant effects of time, group and the time × group interaction were observed (time: $F(3.332, 133.3) = 43.12$, $p < 0.0001$; group: $F(4, 40) = 12.32$, $p < 0.0001$; time × group: $F(13.33, 133.3) = 5.205$, $p < 0.0001$). Sham, saline + distilled water; RES, resveratrol; SDA, saffron-derived antioxidant; PIE, *Passiflora incarnata* L. extract.

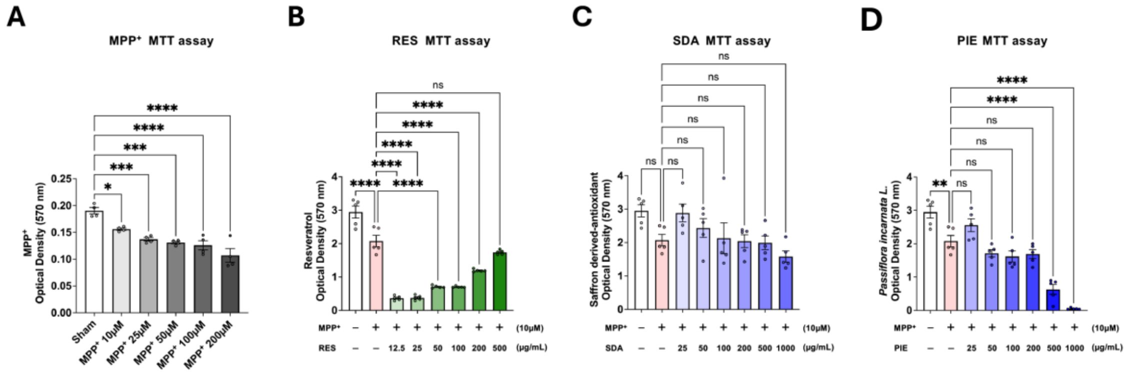

Figure S3. Determination of MPP⁺ and antioxidant treatment concentration ranges. (**A**) MTT assay following treatment with MPP⁺ at 10–200 μM for 24 h to determine the concentration used for subsequent experiments. (**B–D**) MTT assays following MPP⁺ challenge (10 μM) in cells pretreated with increasing concentrations of (**B**) RES (12.5–500 μg/mL), (**C**) SDA (25–1000 μg/mL) or (**D**) PIE (25–1000 μg/mL). A separate 0.5% (v/v) DMSO vehicle-control group was included in the MTT assay to assess the effect of the maximum solvent concentration used during screening; MTT viability did not differ significantly from that of untreated cells (p = 0.0742). Data are mean ± SEM; one-way ANOVA with Tukey's post hoc test. ns, not significant; *, $p < 0.05$; **, $p < 0.01$; ***, $p < 0.001$; ****, $p < 0.0001$. Sham, untreated cells; RES, resveratrol; SDA, saffron-derived antioxidant; PIE, *Passiflora incarnata* L. extract; MPP⁺, 1-methyl-4-phenylpyridinium.

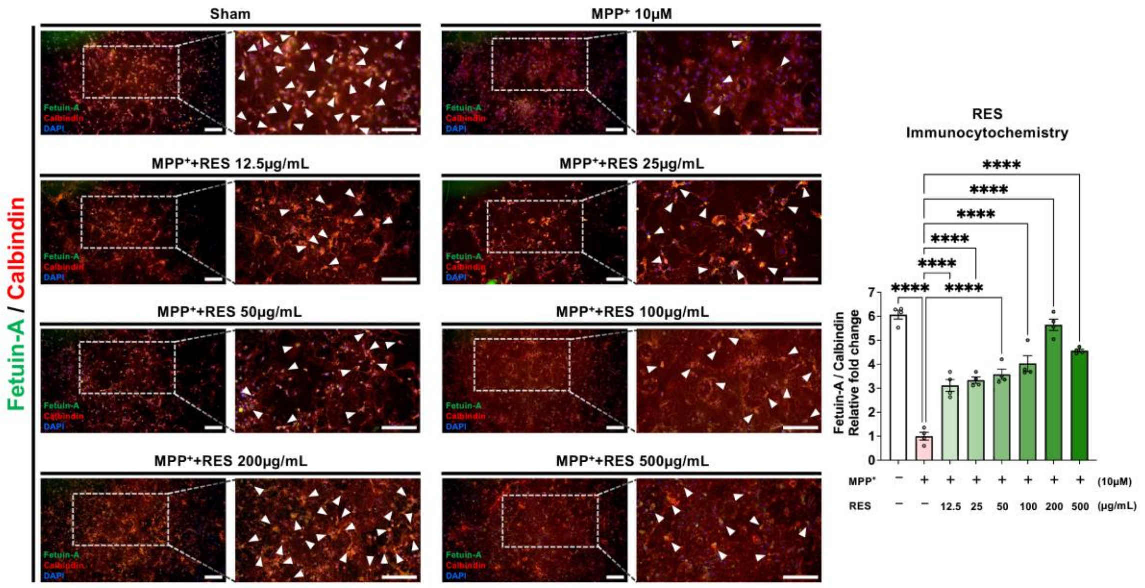

Figure S4. Concentration-dependent effects of RES on Fetuin-A expression. Representative immunocytochemistry for Fetuin-A (green), Calbindin (red) and DAPI (blue) in MPP⁺-challenged primary cerebellar cultures treated with RES at 12.5–500 μg/mL. Images were acquired at 200× and 400× magnification. Boxed regions in the 200× images indicate the areas shown at 400× magnification. Arrowheads indicate representative Fetuin-A⁺/Calbindin⁺ double-positive cells. MPP⁺ was applied at 10 μM. Fetuin-A expression in Calbindin-positive cells was quantified as the fraction of Fetuin-A⁺/Calbindin⁺ double-positive cells among Calbindin⁺/DAPI-positive cells and expressed as relative fold change. Scale bars: 100 μm. Data are mean ± SEM; one-way ANOVA with Tukey's post hoc test. ****, $p < 0.0001$. Sham, untreated cells; RES, resveratrol; MPP⁺, 1-methyl-4-phenylpyridinium.

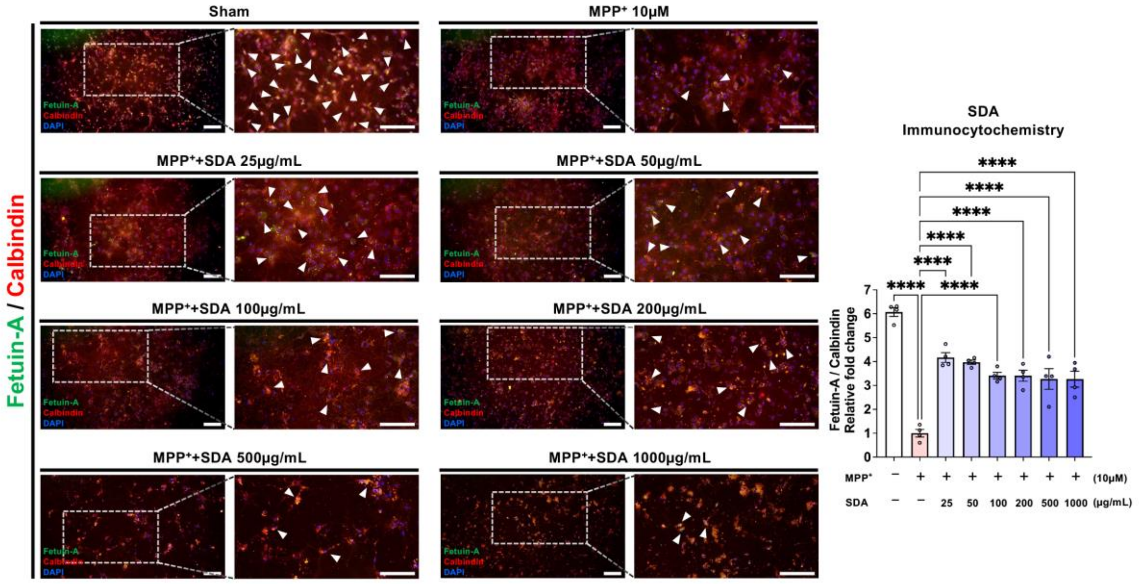

Figure S5. Concentration-dependent effects of SDA on Fetuin-A expression. Representative immunocytochemistry for Fetuin-A (green), Calbindin (red) and DAPI (blue) in MPP$^+$-challenged primary cerebellar cultures treated with SDA at 25–1000 μg/mL. Images were acquired at 200× and 400× magnification. Boxed regions in the 200× images indicate the areas shown at 400× magnification. Arrowheads indicate representative Fetuin-A$^+$/Calbindin$^+$ double-positive cells. MPP$^+$ was applied at 10 μM. Fetuin-A expression in Calbindin-positive cells was quantified as the fraction of Fetuin-A$^+$/Calbindin$^+$ double-positive cells among Calbindin$^+$/DAPI-positive cells and expressed as relative fold change. Scale bars: 100 μm. Data are mean ± SEM; one-way ANOVA with Tukey's post hoc test. ****, $p < 0.0001$. Sham, untreated cells; SDA, saffron-derived antioxidant; MPP$^+$, 1-methyl-4-phenylpyridinium.

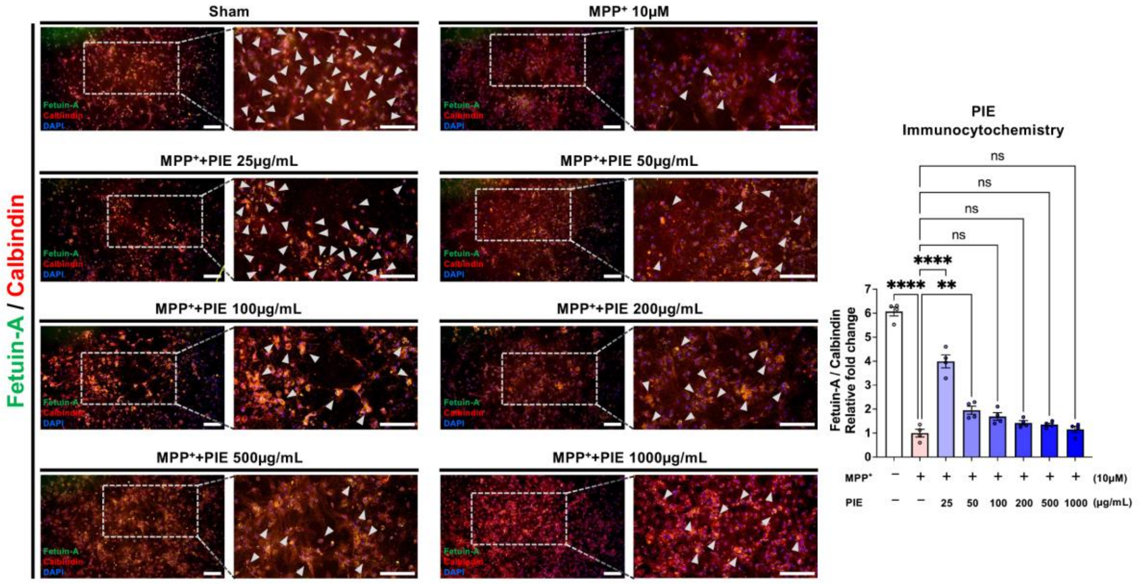

Figure S6. Concentration-dependent effects of PIE on Fetuin-A expression. Representative immunocytochemistry for Fetuin-A (green), Calbindin (red) and DAPI (blue) in MPP$^+$-challenged primary cerebellar cultures treated with PIE at 25–1000 μg/mL. Images were acquired at 200× and 400× magnification. Boxed regions in the 200× images indicate the areas shown at 400× magnification. Arrowheads indicate representative Fetuin-A$^+$/Calbindin$^+$ double-positive cells. MPP$^+$ was applied at 10

μM. Fetuin-A expression in Calbindin-positive cells was quantified as the fraction of Fetuin-A⁺/Calbindin⁺ double-positive cells among Calbindin⁺/DAPI-positive cells and expressed as relative fold change. Scale bars: 100 μm. Data are mean ± SEM; one-way ANOVA with Tukey's post hoc test. ns, not significant; **, $p < 0.01$; ****, $p < 0.0001$. Sham, untreated cells; PIE, *Passiflora incarnata* L. extract; MPP⁺, 1-methyl-4-phenylpyridinium.

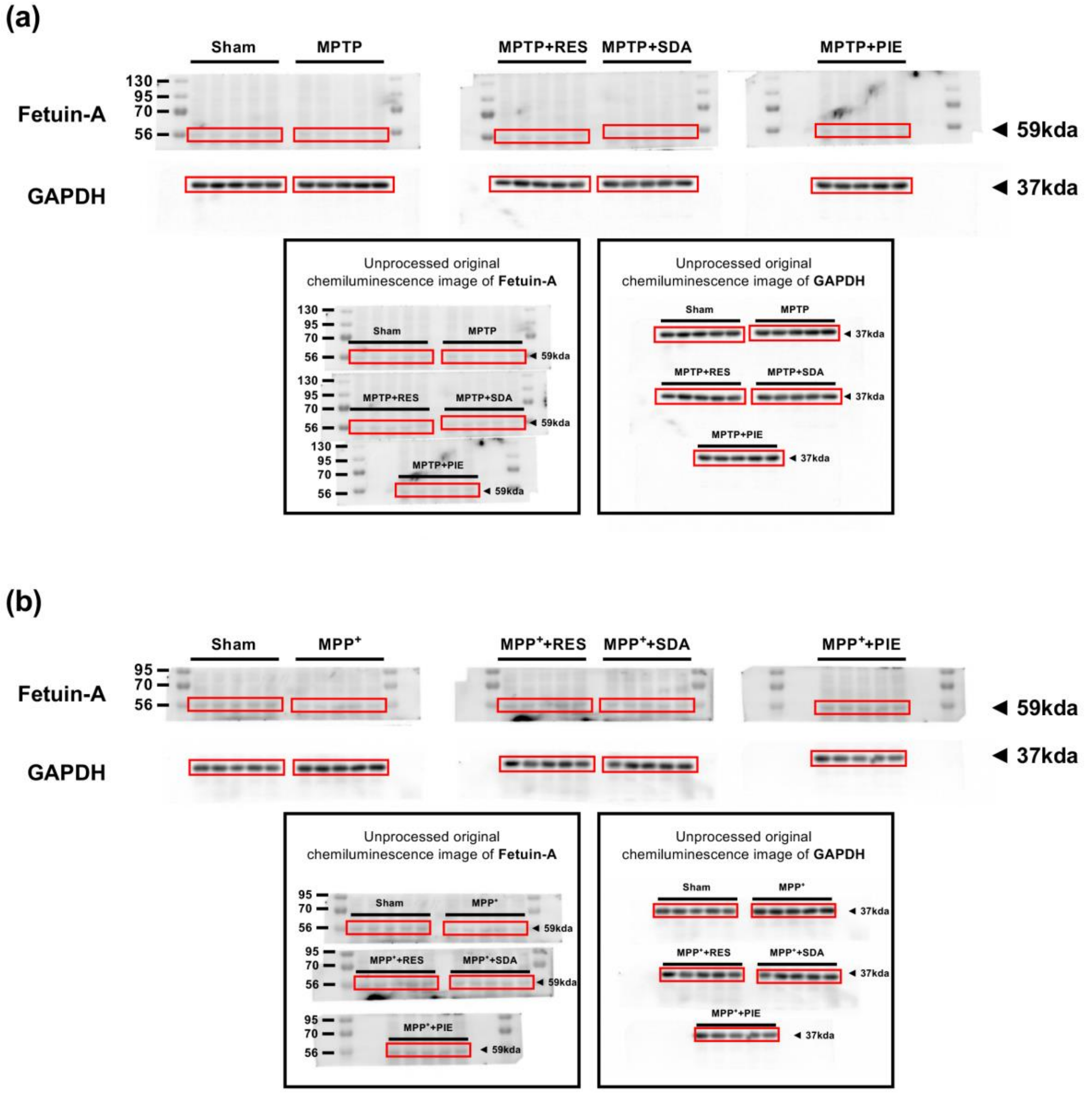

Figure S7. Original Western blot images of the Fetuin-A and GAPDH membrane sections. (a) Original Western blot images corresponding to the Fetuin-A and GAPDH blots presented in Figure 2; (b) original Western blot images corresponding to the Fetuin-A and GAPDH blots presented in Figure 4. Molecular-weight markers are indicated for Fetuin-A (59 kDa) and GAPDH (37 kDa). The membranes were cut horizontally after transfer according to molecular weight, and the corresponding membrane sections were separately probed for Fetuin-A and GAPDH. Molecular weight markers were loaded during electrophoresis. However, the markers were not visible in the original chemiluminescence images of GAPDH. We have therefore provided the original GAPDH images without modification and clarified this limitation. All sample lanes shown in the original images were used in Figures 2 and 4, and their corresponding group identities have been clearly indicated.

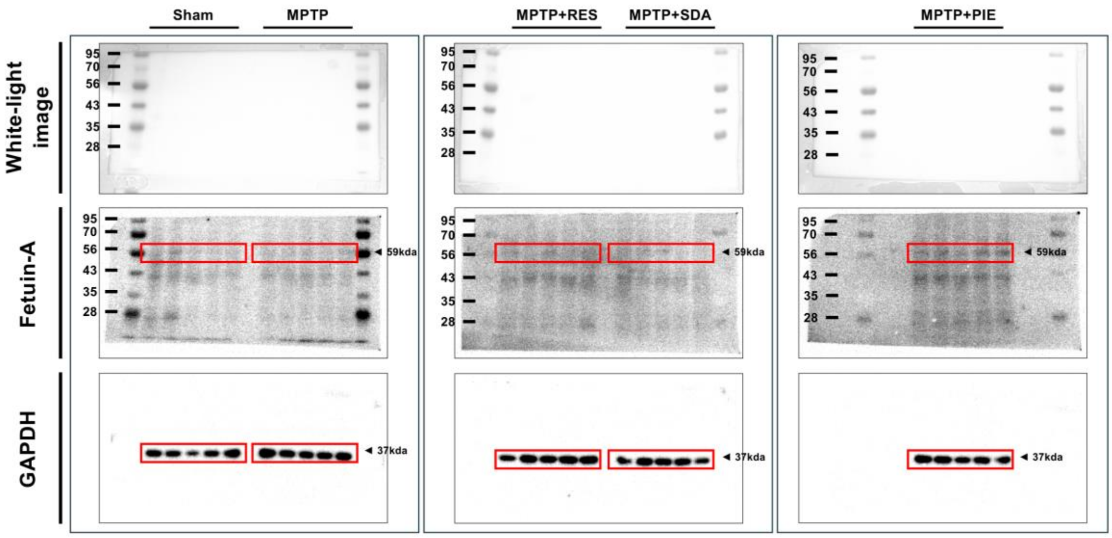

Figure S8. Fetuin-A and GAPDH detected on an intact membrane. Cerebellar lysates from the five groups were separated and transferred as described in Section 2.9, and the membrane was left uncut. The top row shows a white-light image of the intact membrane, with the prestained molecular-weight ladder loaded at both edges. The middle row shows the Fetuin-A signal at 59 kDa and the bottom row the GAPDH signal at 37 kDa, both recorded from that same membrane. The two signals are shown as separate panels because the exposure required for Fetuin-A is considerably longer than that required for GAPDH. The prestained marker bands register in the white-light image and in the longer Fetuin-A exposure, but not in the shorter GAPDH exposure. All panels are shown at the same scale and alignment, so the lanes correspond vertically between rows and the position of the GAPDH bands can be read against the marker positions above. Samples were distributed across three gels, shown here as three membranes. After Fetuin-A detection, each intact membrane was stripped and sequentially reprobed for GAPDH, and separate chemiluminescence exposures were acquired for the two targets. This blot was obtained in a separate experiment using archived lysates and was not quantified. It is provided to document the procedure and is not a quantitative replication of the data shown in Figures 2B and 4B. The Fetuin-A signal is weaker than in the original experiment, which is consistent with the low constitutive abundance of cerebellar Fetuin-A and with the storage time of the samples. Sham, saline + distilled water; RES, resveratrol; SDA, saffron-derived antioxidant; PIE, Passiflora incarnata L. extract.

Table S1. Animal allocation and use for each investigative technique.

Group	Total Animals (*n*)	Behavioral Assessments (*n*)	Histology and Immunostaining (*n*)	Western Blotting (*n*)
Sham	9	9	4	5
MPTP	9	9	4	5
MPTP + RES	9	9	4	5
MPTP + SDA	9	9	4	5
MPTP + PIE	9	9	4	5
**Total**	**45**	**45**	**20**	**25**

Note: All nine animals in each group underwent behavioral assessments before sacrifice. Thereafter, four animals per group were allocated to histological and immunostaining analyses, and the remaining five animals per group were allocated to Western blot analysis. The same four animals were used for all histological and immunostaining analyses, including Nissl staining, immunohistochemistry, immunofluorescence, TUNEL staining, and Fluoro-Jade C staining.
